# Human *Trypanosoma cruzi* infection is driven by eco-social interactions in rural communities of the Argentine Chaco

**DOI:** 10.1371/journal.pntd.0007430

**Published:** 2019-12-16

**Authors:** Maria del Pilar Fernández, Maria Sol Gaspe, Paula Sartor, Ricardo E. Gürtler

**Affiliations:** 1 Universidad de Buenos Aires. Departamento de Ecología, Genética y Evolución, Facultad de Ciencias Exactas y Naturales, Ciudad Universitaria, Buenos Aires, Argentina; 2 Consejo Nacional de Investigaciones Científicas y Técnicas. Instituto de Ecología, Genética y Evolución de Buenos Aires (IEGEBA), Ciudad Universitaria, Buenos Aires, Argentina; 3 Earth Institute, Columbia University, New York, New York, United States of America; 4 Ministerio de Salud Pública del Chaco, Resistencia, Chaco, Argentina; 5 Facultad de Ciencias Exactas, Naturales y Agrimensura, Universidad Nacional del Nordeste, Corrientes, Corrientes, Argentina; Instituto de Ciências Biológicas, Universidade Federal de Minas Gerais, BRAZIL

## Abstract

The transmission of *Trypanosoma cruzi* to humans is determined by multiple ecological, socio-economic and cultural factors acting at different scales. Their effects on human infection with *T*. *cruzi* have often been examined separately or using a limited set of ecological and socio-demographic variables. Herein, we integrated the ecological and social dimensions of human infection risk with the spatial distribution patterns of human and vector (*Triatoma infestans*) infection in rural communities of the Argentine Chaco composed of indigenous people (90% Qom) and a creole minority. We conducted serosurveys in 470 households aiming at complete population enumeration over 2012–2015. The estimated seroprevalence of *T*. *cruzi* prior to the implementation of an insecticide spraying campaign (2008) was 29.0% (N = 1,373 in 301 households), and was twice as large in Qom than creoles. Using generalized linear mixed models, human seropositive cases significantly increased with infected triatomine abundance, having a seropositive household co-inhabitant and household social vulnerability (a multidimensional index of poverty), and significantly decreased with increasing host availability in sleeping quarters (an index summarizing the number of domestic hosts for *T*. *infestans*). Vulnerable household residents were exposed to a higher risk of infection even at low infected-vector abundances. The risk of being seropositive increased significantly with house infestation among children from stable households, whereas both variables were not significantly associated among children from households exhibiting high mobility within the communities, possibly owing to less consistent exposures. Human infection was clustered by household and at a larger spatial scale, with hotspots of human and vector infection matching areas of higher social vulnerability. These results were integrated in a risk map that shows high-priority areas for targeted interventions oriented to suppress house (re)infestations, detect and treat infected children, and thus reduce the burden of future disease.

## Author summary

Chagas disease is one of the main neglected tropical diseases (NTDs) affecting vulnerable communities in Latin America where transmission by triatomine vectors still occurs. Access to diagnosis and treatment is one of the remaining challenges for sustainable control of Chagas disease in endemic areas. In this study, we integrated the ecological and social determinants of human infection with the spatial component to identify individuals, households and geographic sectors at higher risk of infection. We found that human infection was more prevalent in indigenous people compared to creoles and increased with the abundance of infected vectors and with household social vulnerability (a multidimensional index of poverty). We also found that the social factors modulated the effect of the abundance of infected vectors: vulnerable-household residents were exposed to a higher risk of infection even at low infected-vector abundance, and human mobility within the area determined a lower and more variable exposure to the vector over time. These results were integrated in a risk map that showed high-priority areas, which can be used in designing cost-effective serological screening strategies adapted to resource-constrained areas.

## Introduction

Chagas disease is a chronic infection caused by the protozoan *Trypanosoma cruzi*, and is mainly transmitted by triatomine vectors in endemic areas [1]. In spite of regional and intergovernmental vector control (and elimination) initiatives, such as the INCOSUR (“Iniciativa de Salud del Cono Sur”) launched in the 1990s in the Southern Cone countries in South America [2], recent estimates indicate that 62.4% of people infected with *T*. *cruzi* live in INCOSUR countries. Particularly, 30.6% of new cases due to vector-borne transmission in endemic areas occur in Bolivia and Argentina [1], where the Gran Chaco region remains a hotspot for Chagas disease [3,4]. Although in the Argentine Chaco the overall human seroprevalence of *T*. *cruzi* has declined over the last 60 years [5–7], it remains high (27.8–71.1%) in rural communities encompassing creole and indigenous populations [8–16].

Control efforts have traditionally been crafted in a reductionist biomedical approach [17], based on the premise that the sum of information provided by studying each component of the system separately is sufficient to understand disease transmission dynamics [18]. Perhaps as a consequence of this approach, prevention of human *T*. *cruzi* infection has relied almost exclusively on residual insecticide spraying campaigns [19], ignoring the interactions among multiple ecological and social factors and their combined effects on disease transmission. The transmission of zoonotic and vector-borne diseases is an “inherently ecological process” involving intraspecific and interspecific interactions between vectors, pathogens and host populations [20]. However, when human populations are implicated, pathogen transmission dynamics also involve socio-economic, cultural, political, psychological and ethical factors, which pertain to the human dimension of disease [21,22]. Failure to reduce Chagas disease burden in areas such as the Gran Chaco region has prompted to the search of novel interventions based on the Ecohealth or eco-bio-social approaches, which focus on understanding the transmission of infectious diseases as complex socio-ecological systems [17].

In the Gran Chaco, human infection with *T*. *cruzi* usually occurs within sleeping quarters before reaching 15 years of age and is transmitted by *Triatoma infestans* [23–25]. Most studies of human infection with *T*. *cruzi* in endemic areas have focused on the distribution of seroprevalence among demographic subgroups and/or on the effects of vector presence, abundance and *T*. *cruzi* infection status. We hypothesize that the interaction between ecological and social factors would modify the risk of *T*. *cruzi* transmission to humans, which cannot solely be explained by variation in vector indices. Although the association between human *T*. *cruzi* infection and selected socio-demographic factors has been investigated [16,23,26–29], these studies either did not address the combined effects of ecological and social variables due to limited data availability, or only considered a few socio-demographic variables. Human infection has been consistently found to be positively associated with the presence or abundance of domestic animals [16,23,26,27,30], but a less clear association was found between *T*. *cruzi* human infection and house construction quality (i.e., thatched roofs and cracks in the walls): while in some studies human infection increased in poor-quality housing [23,26,29–31], others did not find such association [27,28]. Moreover, no studies have explored how human behaviors such as mobility patterns are associated with human *T*. *cruzi* infection, although a few assessed their effects on house infestation with *T*. *infestans* [32,33].

The joint analysis of the spatial distribution of human and vector infection, as well as the integration of spatially-explicit household-level risk factors, are essential to identify transmission hotspots, create risk maps of *T*. *cruzi* infection, and stratify the affected areas for targeted control [34–36]. For Chagas disease, spatial analysis has been used to investigate the reinfestation process with *T*. *infestans* after community-wide insecticide spraying [33,37–40]; identify factors associated with house infestation [41–43]; evaluate the effects of vector control interventions [44], and generate risk maps at regional and continental levels based on vector distribution [45,46]. By contrast, the spatial component of human *T*. *cruzi* infection has rarely been investigated, and the ones that did it focused on periurban settings [27,47,48].

As part of a broader research and control program on the transmission of *T*. *cruzi* in rural communities of the Argentine Chaco [16,33,41,42,49–53], previous studies have found a strong association between vector indices and social determinants [33,41,54]. In one of these studies, we constructed a social vulnerability index using multiple correspondence analysis (MCA) to synthesize the multiple dimensions of poverty–a method that has been widely used for this purpose in low and middle income countries [55]–and a domestic host availability index (a key ecological factor); both indices were positively associated with the abundance of infected domiciliary *T*. *infestans* [54]. Herein, we focus on human *T*. *cruzi* infection at individual, household and community levels, and assessed its association with vector-related factors, social determinants, and their interactions to shed light into the processes associated with the vector-borne transmission of *T*. *cruzi* to humans. Specifically, we investigated the effects of household-level vector indices, human demographic factors (including mobility patterns), household socio-economic status and domestic host availability on human *T*. *cruzi* infection status derived from cross-sectional serosurveys. At a community level, we evaluated the spatial correlation between human and vector infection, host availability and social vulnerability. We also integrated the evidence provided by the individual-based risk model with the spatial component to derive community-level risk maps of human infection, with an emphasis in children, which represent the target demographic group for diagnosis and treatment of *T*. *cruzi* infection.

## Methods

### Study area

**Fig 1:**
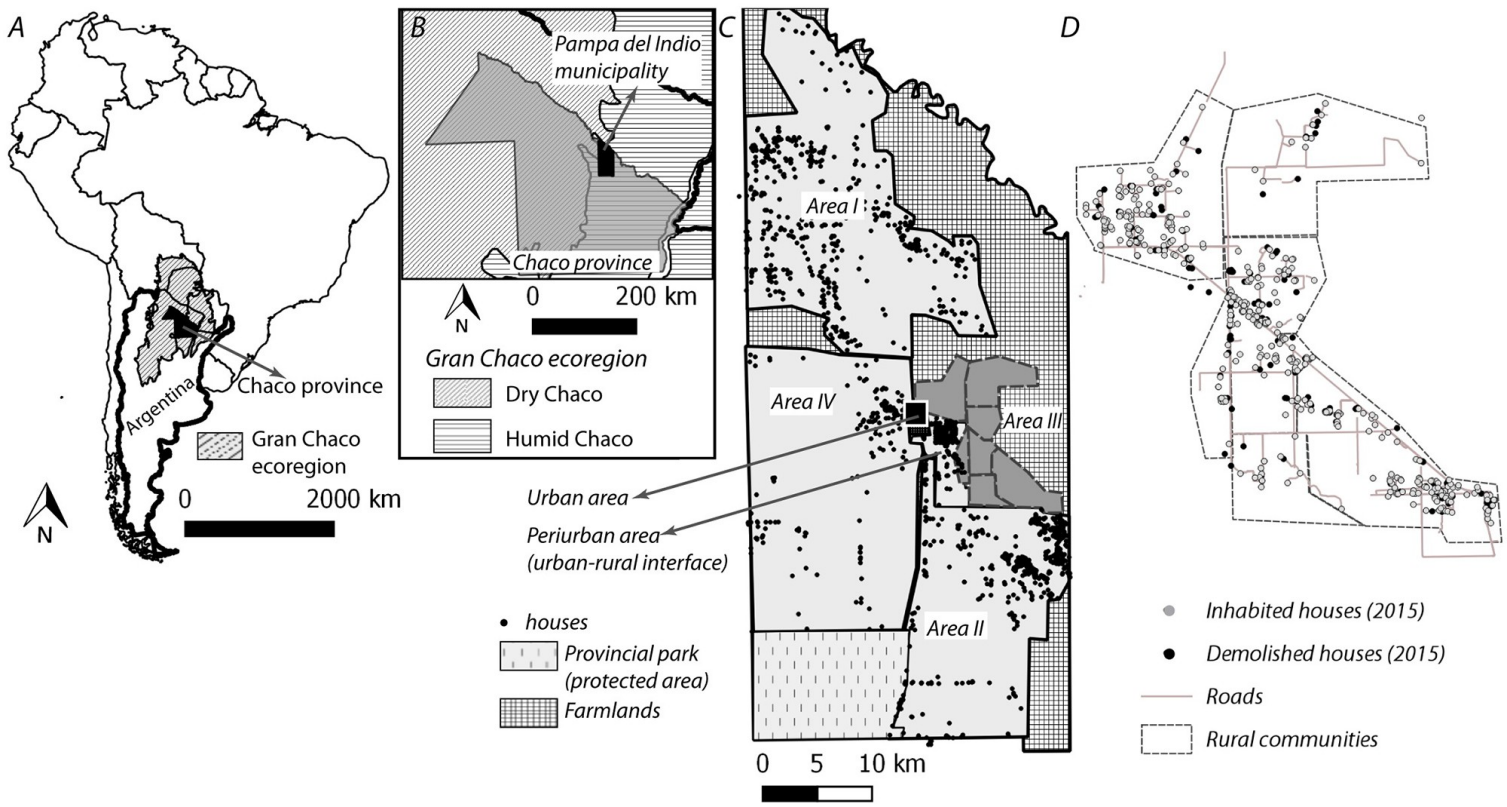
Map of the study area. (A) Location of Chaco Province, northeastern Argentina, within the Gran Chaco ecoregion. (B) Location of Pampa del Indio municipality within Chaco Province. (C) Urban, peri-urban and rural areas (I-IV) of the municipality. (D) Study area (Area III). The maps were created in QGIS 2.18.11. based on publicly available data (i.e., political boundaries, [56]) and data collected in a previous study [33].

This study was conducted in a rural section of Pampa del Indio municipality (25° 55’S 56° 58’W), Chaco province, Argentina, denominated Area III, which encompassed three contiguous large communities (Cuarta Legua, Pampa Grande and Pampa Chica), and other smaller ones [41] (Fig 1). Prior to 2008, vector control actions had been sporadic and partial; the last community-wide insecticide spraying campaign took place in 1997–1998, and the last sprays registered prior to the baseline survey were carried out in a few houses in 2006 and July 2008 [41].

This area was subjected to a vector control and Chagas disease research program initiated in 2008 with a follow-up period of 7 years as of 2015, as described in two previous studies which focused on the eco-bio-social determinants of house infestation [41] and re-infestation [33] with *T*. *infestans*, respectively (S1 Fig). In October 2008, a baseline vector survey found that a third of the inhabited houses were infested with *T*. *infestans*, mainly within human sleeping quarters; virtually all (93.4%) houses were sprayed with insecticides immediately after [41]. During the 2008–2015 vector surveillance phase we conducted annual triatomine surveys and selectively sprayed with insecticide the few foci detected. This strategy reduced house infestation to <1% during 2008–2012, and no infested house was found in 2015 [33].

The population in the area monotonically increased from 2,392 people and 407 inhabited houses in 2008 to 2,548 people and 587 houses in 2015 [54]. The population was composed of two ethnic groups; 93.1% of the residents self-identified as Qom people and the remaining minority as creoles [54]. A household was defined as all the people who occupy a housing unit, including related and nonrelated family members [57]. The housing unit (or house compound) included one or more domiciles (i.e., sleeping quarters) and associated peridomestic structures.

### Study design

This study complied with STROBE recommendations for observational studies [58] (S1 Text). We integrated vector and epidemiological information collected during the 2008–2015 period during the annual vector and household surveys as described in detail elsewhere [33,41,54], with human infection data collected during 2012–2015 (S1 Fig). At each annual vector survey, the location of the domicile was georeferenced with a GPS receiver (Garmin Legend) and the demographic information of the household updated. The household surveys included relevant epidemiological data such as number, type and construction materials of the different sites within each housing unit, refuge availability for the vector, the number and resting places of domestic animals, number of house occupants, and their ages and ethnicity. In 2012 and 2015, we also carried out a census of the population, which included several variables identified as social determinants of health and relevant to NTD transmission, such as educational level, income source, household mobility, access to health services, among others [54]. Response rates were high (>90%) in all surveys [33].

Four years after the community-wide insecticide spraying campaign, under a very low risk of vector-borne transmission (<1% of infested houses and bug infection) [33], we conducted a series of serosurveys aimed at total population coverage (including individuals older than 9 months old) during the 2012–2015 period. Using a participatory approach, we had meetings with the local population to discuss project tasks and coordinate activities with local healthcare agents and hospital personnel [50]. During 2012–2013, people were recruited passively through the radio, schools and word of mouth; venipuncture was done at schools and at the primary health posts of each large community to draw a blood sample of 3–7 ml. Recruitment was done actively during April 2015 and blood draws were conducted house-to-house coupled to the vector survey. The protocol employed during the serosurveys is described in detail elsewhere [16,50].

### Ethics statement

This study complied with the ethical principles included in the Declaration of Helsinki (Ethical Committee "Dr. Carlos A. Barclay", Protocol ref. TW-01-004). All adult subjects provided written informed consent prior to participation in the serosurveys, and a parent or guardian of any child participant younger than 14 years old provided informed consent on the child’s behalf. Participants aged between 14 and 18 years old, provided their written informed consent along with consent from a parent or guardian. Informed consent forms were provided in Spanish and translated to Qom language (*Qomlactaq*) orally by primary healthcare agents when needed. Oral consent was obtained for household surveys, including triatomine searches, as approved by the IRB and recorded in the data collection sheets as described [41].

### Serodiagnosis of human infection

Blood samples were transported to the local hospital where serum samples were obtained by centrifugation at 3,000 rpm for 15 min within the same day. Each serum was aliquoted in triplicate vials and stored at -20°C. Serum samples were tested using two different ELISA tests based on conventional (Chagatest, Wiener) and recombinant antigens (ELISA Rec V3.0, Wiener) as described elsewhere [50]. Discordant results between both ELISAs were resolved via an indirect immunofluorescence antibody test conducted at the reference center for Chagas disease serodiagnosis (Instituto Nacional de Parasitología Dr M. Fatala Chaben). A person was considered seropositive (i.e., infected) if at least two tests were reactive. The serosurveys’ results were informed to the participants within 8 weeks of the blood draw, and follow-up of *T*. *cruzi*-seropositive people was done by physicians from the local hospital as described elsewhere [50], including treatment with benznidazole or nifurtimox of seropositive patients younger than <18 years old (y.o.) and those seropositive adults who specifically requested it.

### Vector-related indices

All triatomines collected at the baseline vector survey in 2008 were identified taxonomically and the individual infection status with *T*. *cruzi* was determined by microscope examination of feces [41] or by molecular diagnosis using kDNA-PCR [59], achieving a coverage of 60% of all infested houses (S1 Fig). The occurrence of domiciliary infestation with *T*. *infestans* was determined by the finding of at least one live triatomine (excluding eggs) through any of the vector collection methods used (i.e., timed-manual searches, bug collections during insecticide spraying operations, and householders’ bug collections). The relative abundance of domiciliary *T*. *infestans* was calculated only for infested houses as the number of live bugs collected by timed-manual searches per 15 min-person per site, as described [41]. The same procedures were used to determine the occurrence and abundance of *T*. *cruzi*-infected *T*. *infestans* in the domicile.

### Epidemiological data pre-processing

Human seropositivity data (collected over 2012–2015) were back-corrected to 2008 in order to analyze human infection prior to the community-wide insecticide spraying campaign when vector-borne transmission was still active, and given the virtual absence of house infestation and of infected triatomines over 2008–2015 [33]. We did this by correcting the age recorded at the time of the serosurveys (using the birth date) and assigning the corresponding age at the baseline survey in 2008; this allowed us to compute a more accurate length of exposure to vector-borne infections by subtracting the time elapsed since the baseline vector survey to the age recorded at the subsequent serosurveys. We also identified whether the person was living in the area in 2008 and their specific residential location by using the demographic data collected in household surveys over 2008–2015 and the location of each household over time. The 2012–2015 detailed census data was also used to determine their mobility patterns: Individuals were classified as: non-movers (individuals whose house remained at the same geographical location), movers (if they moved within the study area), or as in-migrants or out-migrants (to or from outside the study area, respectively, including individuals coming from or leaving to a different section within Pampa del Indio municipality), as described elsewhere [54]. If the in-migrants had resided in the study area prior to 2008, we classified them as return migrants.

Regarding the socio-demographic variables collected in 2012, they were back-corrected to 2008 whenever possible, assuming similar socio-economic conditions prevailed over 2012 and 2008 [41]. This back-correction consisted of identifying the specific residential location of the household within the study area as of 2008 and matching it with the vector and other epidemiological data collected at baseline. In a previous study focused on the social determinants of health associated with Chagas disease in these communities [54], we used multiple correspondence analysis to construct two surrogate indices (the social vulnerability index and the host availability index) based on the data obtained from the household surveys. A detailed description of the indices, their rational and construction is found elsewhere [54]. The social vulnerability index estimated for 2008 households included characteristics of the domiciles (refuge availability, presence of cardboard roofs and/or mud walls, time since house construction, and domestic area), and household socio-economic and demographic characteristics (overcrowding, goat-equivalent index and educational level). This index aimed at summarizing the multiple interrelated socio-demographic variables associated with socio-economic position, and proved useful to capture between-household heterogeneities in socio-economic status in the context of structural poverty [54]. The host availability index in domiciles summarized the number of potential domestic hosts of *T*. *infestans* (i.e., adult and child residents, chickens nesting indoors, and the total number of dogs and cats in the household), and whether the latter rested within or in close proximity to the domicile as an additional binary variable. The host availability index relied on a preliminary analysis showing that the household abundance of domestic animal hosts was positively correlated with the number of human residents [54].

### Data analysis

We first estimated the baseline prevalence of human *T*. *cruzi* infection by age and gender, followed by a descriptive analysis of the household distribution of human infection, and its bivariate association with vector abundance and the relevant socio-economic indices [54]. We hypothesized that human infection in children younger than 15 years old would be more closely associated to vector abundance compared to the total population, and that the number of cases per household would be more closely associated to vector indices and host availability since humans are also a source of infection for triatomines and increase the household transmission of *T*. *cruzi*. In the case of children, we also analyzed the association between human mobility patterns recorded over 2012–2015, and infection prevalence at a household level to test the hypothesis that movers would have less stable exposure to the vector compared to non-movers. We then analyzed the spatial distribution pattern of human infection (occurrence and number of cases per household) at a community level, hypothesizing that even though human infection is mainly affected by household-level variables, spatial aggregation of cases at a higher spatial scale might occur because of dispersal of infected vectors or human mobility between households (see spatial analysis section below). We finally integrated the ecological and socio-demographic variables in an infection risk model for the total population and for children to test our general hypothesis of eco-social interactions, and interpolated the results of the risk model to create a risk map (see infection risk model section below).

For each variable we checked whether the missing values were missing completely at random by building a dummy variable (missing and non-missing values) and analyzing its association with any another independent variable in the data set using multiple logistic regression models. Most of the variables with missing values (collected through household surveys) were missing completely at random, except for the social vulnerability index in 2008, in which the missing data corresponded to households that had moved or out-migrated by 2012 (the year when educational level and overcrowding data were collected), and thus were not missing at random. Missing data for human infection per household were also biased towards adult males (who refused to participate more often than other demographic groups), and movers, as we were not able to assign their 2008 residential location to all of them. We used listwise deletion of cases with missing data (28.6% of all records) to achieve a complete data set because the available data were insufficient to build a robust data imputation model. We excluded from the analysis 197 people tested who resided elsewhere but were visiting the study area at the time of the serosurveys. Throughout the text, minors younger than 15 y.o. are referred to as children for simplicity (S1 Fig).

For all proportions, 95% confidence intervals (CI_95_) were estimated using the Agresti and Coull method if sample sizes were greater than 50, and the Wilson method for smaller sample sizes [60]. We used χ^2^ tests for bivariate analysis of categorical variables; generalized linear models (GLM) [61] with logit link function when the outcome of interest had a binary distribution, and a negative binomial GLM (link function: log) when analyzing count data, such as the number of seropositive people. In the case of binary response variables, the measure of association between outcome and explanatory variables was expressed as Prevalence Odds Ratios (POR), and in the case of count data as Prevalence Rate Ratios (PR), which are preferred for cross-sectional studies and type of analysis employed [62]. Negative binomial regression was preferred to Poisson regression given the overdispersed distributions [63]. Mixed-effects models (GLMM) were also considered when individual data were analyzed to account for possible household-related random effects [16,26]. All analysis were implemented in Stata 14.2 [64] and R 3.2.3 (lme4 and car packages) [65].

### Infection risk models

The outcome of interest was the person’s seropositivity status for *T*. *cruzi* (yes/no). In this study we referred to “infection risk” as the probability of an adverse outcome, thus the PORs represent the relative risk of seropositivity comparing exposed and unexposed people [66]. The infection risk model was estimated for the entire population and for children separately, hypothesizing that infection was acquired before 15 years of age and children would be more closely associated with the conditions measured at baseline and during the study period. In the model for the total population we included age, gender, ethnicity, and household-level variables: the relative abundance of *T*. *cruzi*-infected *T*. *infestans* per unit of catch effort in the domicile, the social vulnerability index, host availability index, and the number of seropositive co-inhabitants. The model for child infection also included the mother’s seropositivity status, and whether any recent insecticide spraying occurred in 2006–2008 prior to the baseline vector survey. We also evaluated the interaction between vector abundance, the social vulnerability and host availability indices, and retained in the model the ones that had a significant effect. To test aggregation of infection at a household level, we used a log-likelihood ratio test to compare a GLMM model (logit link function) considering the household as a random variable and a simple GLM model without considering the household as an explanatory variable.

We used an information theoretic approach and Akaike’s information criterion (AIC) to identify the best-fitting models given the data collected, and a multimodel inference approach to account for model selection uncertainty [47,48] using the MuMin R-package. This approach was used to identify the ecological and social factors associated with house infestation, as described elsewhere [41]. PORs and their 95% confidence intervals were calculated from model-averaged coefficients. The relative importance (RI) of each variable is defined as the sum of Akaike weights in each model in which the variable is present; RI takes values from 0 to 1. Multicollinearity was assessed by the Variance Inflation Factor (VIF), and model fitting for the logistic regression was assessed by the Hosmer-Lemeshow goodness of fit test and the Receiver Operator Curve (ROC), including the area under the ROC (AUC) [65]. To assess model sensitivity and specificity, we employed an optimal threshold value that minimized the sum of error frequencies [67]. This value was obtained by finding the maximum sum of sensitivity and specificity for all threshold values t (sens(t)+spec(t)) using the pROC R-package [68]. Additionally, the H-index was employed as an alternative measure of model classification performance [69]. This aggregated index of performance takes into account misclassification costs, which seek to quantify the relative severity of one type of error over another. We considered that misclassifying a seropositive person was more costly than misclassifying a seronegative person. A higher H-index indicated better performance. The H-index allows comparisons of models across different datasets and classifiers and was implemented using the R-package “hmeasure” [69].

### Spatial analysis and risk maps

Global point pattern analysis (univariate and bivariate) were performed for human and vector infection using the weighted K-function implemented in Programita [36]. Random labeling was used to test the null hypothesis of random occurrence of events among the fixed spatial distribution of all households. We used quantitative (e.g. number of infected people) and qualitative labels (e.g., occurrence of at least one infected person) for each household as previously described [54]. Monte Carlo simulations (n = 999) were performed and the 95% ‘confidence envelope’ was calculated with the 2.5% upper and lower simulations. In the case of the qualitative labels we present the L(r) Ripley statistic by the distance (r) and for the quantitative labels we present the rho(r) mark variogram; the latter indicates whether neighboring households present similar mark values (a lower rho value means more similar values) evaluated at each distance r [36]. In the case of bivariate analysis, the values are normalized and the rho value represents how the marks vary together (a lower rho value means a positive correlation between marks).

Local spatial analysis on the abundance of (infected) vectors and number of infected people were performed using the G* statistic implemented in PPA [34]. The selected cell size was 200 m (assuming that each household had at least three neighbors at the minimum distance of analysis), and the maximum distance was set at 6 km (i.e., half of the dimension of the area). We created heatmaps (i.e., density maps) to visualize the spatial aggregation of the variables using a kernel density estimation algorithm within a radius of 200 m as implemented in QGIS 2.18.11. Risk maps using the 2008 household locations were obtained by interpolating the predicted probabilities from the risk model. The geographical coordinates of each housing unit were transformed to preserve the privacy of the households involved in this study, as described elsewhere [41].

## Results

We tested 1,929 people that resided in the study area at the time of the serosurveys conducted over 2012 and 2015. Passive recruitment during 2012–2013 at local schools and primary health posts led to a coverage of 47.8% of the local population; this increased to 77.0% during 2015, when active recruitment of participants was coupled with vector surveys (S1 Fig). Diagnosis coverage was highest in school children (80.9%) and adult women (76.8%) (S2 Fig).

**Fig 2:**
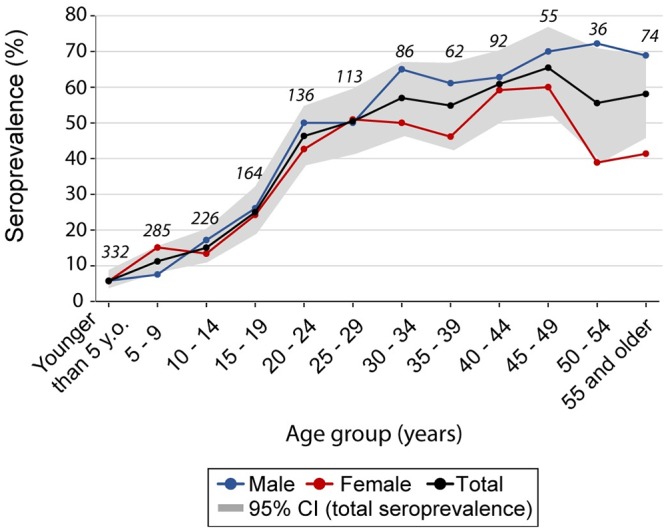
Seroprevalence of *T*. *cruzi* infection per 5-year age group and gender for the human population in 2008, prior to a community-wide insecticide spraying campaign, Pampa del Indio, Argentina. The numbers above the lines indicate the total number of people serologically tested.

Exposure to infected vectors in the study area was negligible during the vector surveillance period spanning from 2009 to2015 (S2 Text). We were able to assign the local residency status as of 2008 (i.e., their house identification code in 2008) to 82.6% of the people tested. The overall seroprevalence estimated for 2008 (i.e., at the baseline vector survey) was 29.0% (CI_95_ = 26.7–31.4, n = 1,373). It increased significantly with age from 5.7% in children younger than 5 y.o. to 25% in teenagers (14 to 19 y.o.), then jumped to 50% in young adults (20 to 29 y.o.), and remained around 60% in older adults (Fig 2). Although females had a lower overall seroprevalence rate than males (26.5 vs 31.6%; χ^2^ test, df = 1, p = 0.04), this difference was more evident in adults (Fig 2) and was not significant after adjusting for age (S1 Table). The seroprevalence for Qom people exceeded substantially that observed for creoles (29.7 vs 18.7%; χ^2^ test, df = 1, p = 0.02) (S1 Table).

### Household infection and domiciliary infestation

We found at least one *T*. *cruzi*-seropositive person in 71.3% of the 301 households in which at least one member had been tested (77% of all households existing in 2008). For children, that proportion dropped to 16.7%. The occurrence of at least one seropositive person per household was not significantly associated with the abundance of *T*. *cruzi*-infected *T*. *infestans* per unit effort in the domicile (POR = 1.5, CI_95_ = 0.8–3.0; p = 0.2). In contrast, the occurrence of at least one infected child was positively and highly significantly associated with the abundance of infected vectors (POR = 2.8; CI_95_ = 1.6–4.9; p < 0.001).

The seroprevalence of *T*. *cruzi* was higher in local residents who reported past exposure to *T*. *infestans* at the time of the serosurveys (35.3 vs 15.0%, χ^2^ test, df = 1, p < 0.001). There was a slight agreement between the 2008 house infestation status and reported past exposure to triatomines (kappa index = 0.2; p < 0.001). Although 79% of residents in an infested house reported past exposure to the vector, a considerable proportion (54%) of those who inhabited non-infested houses also reported past exposures. Therefore, infection prevalence adjusted for age, gender and ethnic group and clustered by household was two-fold higher among residents of infested houses as determined in the 2008 (baseline) vector survey (POR = 2.3, CI_95_ = 1.4–3.9, p < 0.001), but the effect of infestation on infection prevalence was marginally significant when the self-reported past exposure was considered instead (POR = 1.5, CI_95_ = 1–1.2, p = 0.6) (S2 Table). In children, the infection prevalence if living in infested houses was almost four-fold higher (POR = 3.7, CI_95_ = 1.3–10, p = 0.01) even after adjusting for the mother’s seropositivity status, while the reported past exposure had no significant effect (POR = 0.9, CI_95_ = 0.4–2.4, p = 0.9) (S2 Table). When vector abundance per unit effort was considered, the infection prevalence in children increased 42% per 5 additional *T*. *infestans* collected in the domicile (POR = 1.42, IC_95_ = 1.2–1.7, p = 0.001).

### Household mobility and human infection

Movers (as registered in 2012–2015) had a significantly higher risk of residing in a *T*. *infestans*-positive house in 2008 than non-movers after adjusting for age, gender and ethnic group (POR = 1.7; CI_95_ = 1.3–2.1; p < 0.001). These findings also proved valid in children, whose infection status is more closely associated with recent domiciliary infestation. However, child infection was not directly associated with their mobility pattern (S3 Table). Instead, household mobility acted as a modifying factor of the association between domiciliary infestation and child infection: while the risk of being seropositive increased significantly with house infestation among non-mover children, no significant association was observed between house infestation and infection status among mover children (Table 1, S3 Fig). The effects of infestation on child infection risk decreased by 90% in movers compared to non-movers (POR_interaction_ = 0.1; CI_95_ = 0.01–0.99; p < 0.001, S3 Table).

**Table 1:** Prevalence Odds Ratios (POR) of the effects of the interaction between mobility patterns and house infestation on child seropositivity for *T*. *cruzi infection* in 2008, Pampa del Indio, Argentina. The interaction analysis was included in a generalized linear mixed model clustered by household and adjusted for age, gender, ethnic group and the mothers’ seropositivity.

	POR (CI_95_)	
Mobility pattern	Non-infested house	Infested house
Non-mover	1	6.1 (1.9–19.1)*
Mover	1.8 (0.4–8.5)	1.0 (0.0–161.9)
Out-migrants	2.2 (0.2–20.0)	0.0 (0.0-inf)
Return migrants	0.0 (0.0-inf)	0.3 (0.0-inf)

POR: Prevalence odds ratio; CI_95_: 95% confidence interval; inf: infinity

** p < 0.001;

* 0.001 ≤ p ≤ 0.05;

^~^ 0.5 < p < 1

### Household socio-demographic determinants of human infection

**Fig 3:**
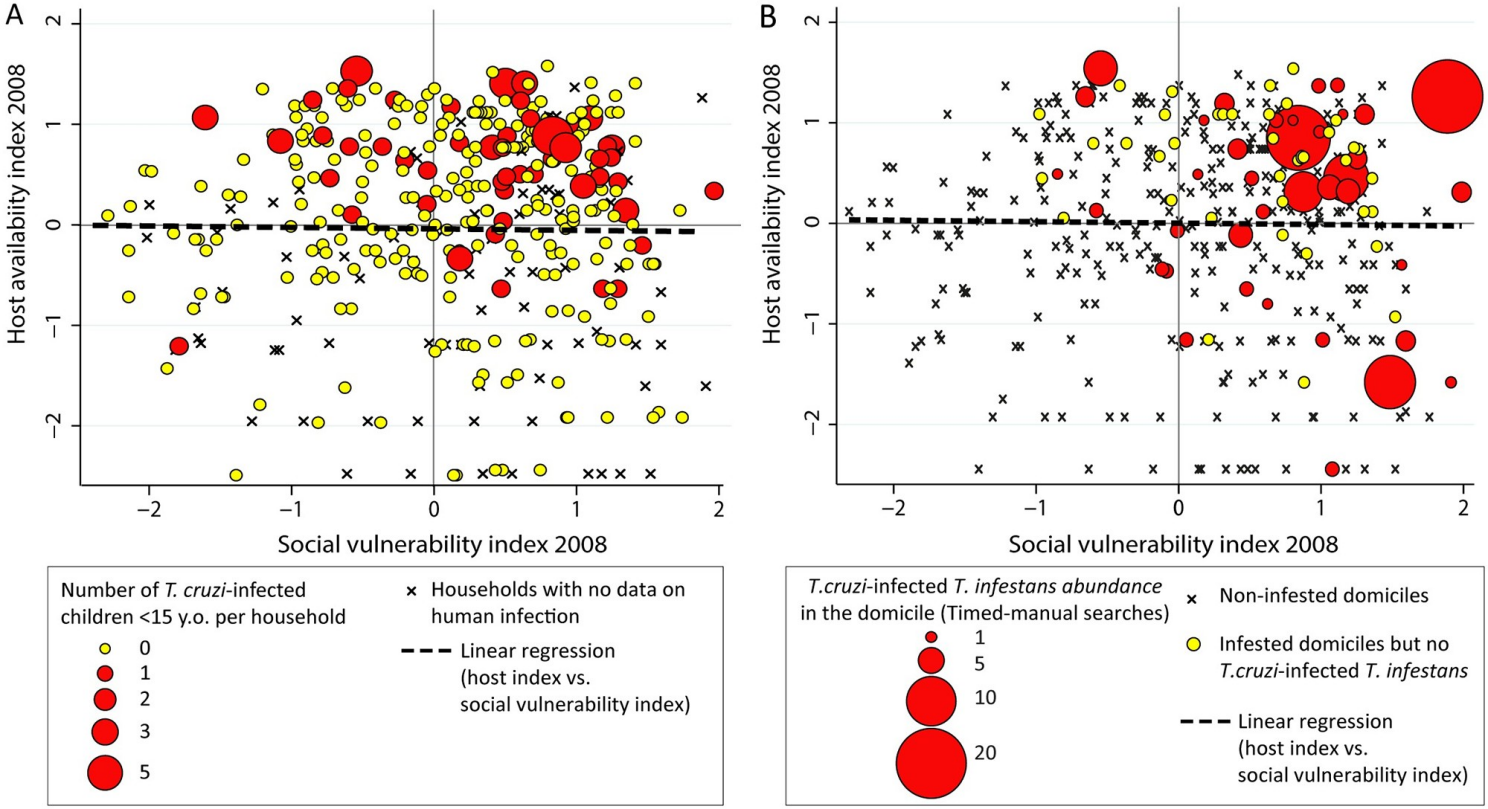
Multivariate relationship between household-level host availability, social vulnerability and vector indices [54] (A) and child seropositivity for *T*. *cruzi* infection (B), Pampa del Indio, Argentina.

The occurrence of at least one seropositive person per household was positively associated with social vulnerability (POR = 1.5; CI_95_ = 1.2–1.8; p = 0.001) and was independent of host availability (POR = 1.3; CI_95_ = 0.9–1.7; p = 0.1). The household presence of at least one seropositive child occurred more frequently in houses with high social vulnerability (POR = 1.4; CI_95_ = 1.0–2.0; p = 0.05) and high host availability (POR = 2.1; CI_95_ = 1.4–3.4; p < 0.001) (Fig 3A). In both cases, the interaction terms between socio-demographic variables were not significant, indicating no effect modification in the multiplicative odds ratio scale. The occurrence and abundance of *T*. *cruzi*-seropositive children were similar to the distribution of infected domiciliary *T*. *infestans*, which also increased with host availability and social vulnerability (Fig 3A and 3B).

### Spatial distribution of human infection and house infestation

**Fig 4:**
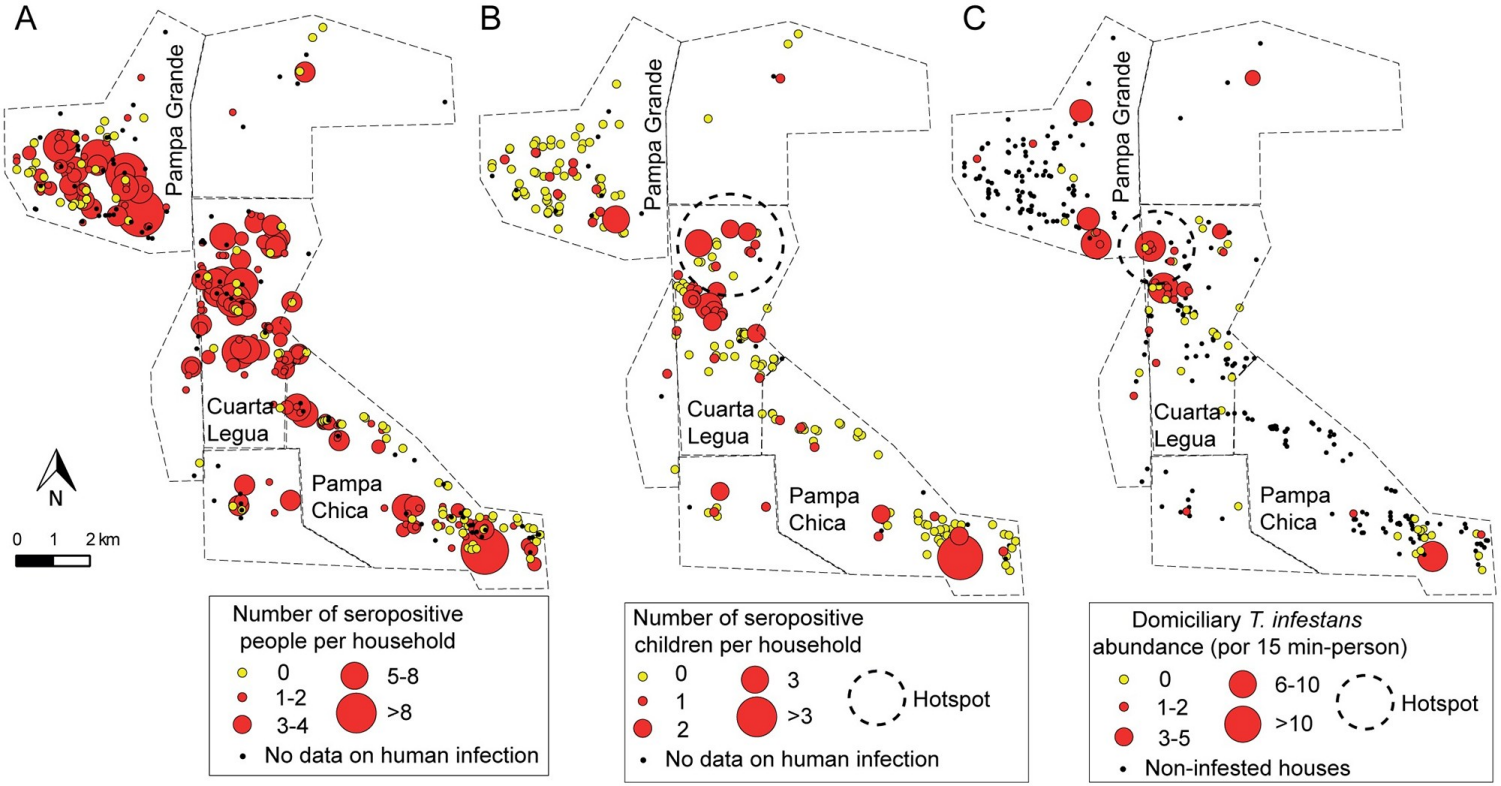
Distribution of the household number of *T*. *cruzi*-seropositive people (A) and children (B), and the relative abundance of infected *T*. *infestans* per unit of effort (C) prior to community-wide insecticide spraying in 2008, Pampa del Indio, Argentina. The dashed circles represent the hotspot found by local spatial analysis. The maps were created in QGIS 2.18.11. based on the data collected within the scope of this study (S4 Table).

Human infection was spatially aggregated in 2008 despite the widespread occurrence of at least one *T*. *cruzi*-seropositive person per household (Fig 4A). The global spatial analysis indicated that households with at least one seropositive person were aggregated at a scale between 2 and 6 km (S4A Fig), implying that up to 2 km (i.e., within each community) the spatial distribution was not significantly different from a random pattern. This result is consistent with the differences among communities in the proportion of households with at least one seropositive person: 84.6% in Cuarta Legua, 69.4% in Pampa Grande and 56.8% in Pampa Chica (χ^2^ test, df = 2, p < 0.001). When the number of seropositive people per household was considered, we did not find a significant spatial correlation between neighboring houses at any scale. Although the trend shown by the variograms indicated similar numbers of seropositive people between neighboring households, this pattern was not significantly different from a random distribution (S4B Fig).

The proportion of houses with at least one seropositive child in 2008 also varied significantly between communities (χ^2^ test; df = 2; p < 0.01). The highest proportion was found in Cuarta Legua compared to the other communities (32.9% vs. 14.5–13.8%, respectively), but no spatial aggregation was found globally (S4C Fig). The number of infected children per household did not show a spatial correlation between neighboring households either (S4D Fig). However, local spatial analysis found a hotspot of child infection located in Cuarta Legua at a scale between 1 and 2.6 km (Fig 4B). This hotspot was no longer detected in 2012–2015, nor did the spatial aggregation per community for the total population.

The occurrence of at least one *T*. *cruzi*-infected *T*. *infestans* in the domicile was globally aggregated at all spatial scales when considering all houses (S4E Fig), but it did not differ from a random distribution when only infested houses were considered (S4F Fig). Nonetheless, a hotspot of domiciliary infected vectors was found in Cuarta Legua at a spatial scale between 0.2–1.8 km, which involved 6 houses with at least one infected vector (Fig 4C). This hotspot coincided partially with the child infection hotspot, but no significant correlation was found between the global human and vector infection patterns (S4G and S4H Fig).

**Fig 5:**
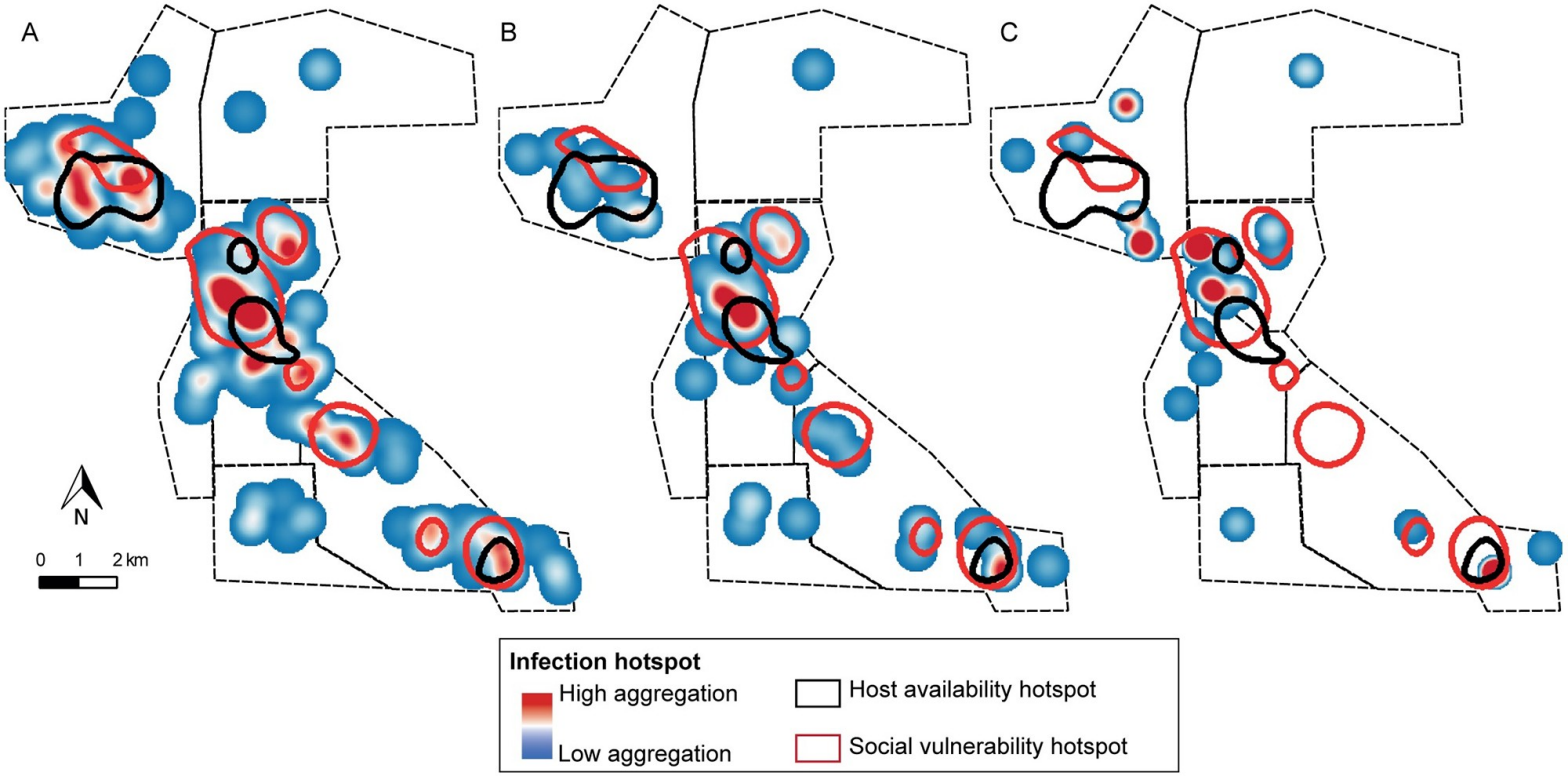
Heatmaps of the household number of *T*. *cruzi*-seropositive people (A) and children (B), and the relative abundance of *T*. *cruzi*-infected *T*. *infestans* per unit of effort (B) in 2008, prior to a community-wide insecticide spraying campaign, Pampa del Indio, Argentina. The areas of qualitative aggregation (as determined by heatmaps) of social vulnerability and host availability are also shown. The maps were created in QGIS 2.18.11. based on the data collected within the scope of this study (S4 Table).

The heatmaps show that the areas of high social vulnerability coincided with the qualitative and quantitative hotspots of human (total and children) and vector infection (Fig 5). However, no significant spatial correlation was found between human or vector infection and socio-demographic variables (social vulnerability and host availability) (S5 Fig).

### Human infection risk model

Human infection was significantly clustered by household after adjusting for the abundance of infected vectors, household social vulnerability and host availability (β_household_ = 1.3, CI_95_ = 1.02–1.7, log-likelihood ratio test, p < 0.001). When we included the number of seropositive co-inhabitants, the random effect of the household was nil and no significant differences were found between the GLMM and the respective GLM model (log-likelihood ratio test, p = 1), indicating that all variables included in the model accounted for the differences among households.

The prevalence of *T*. *cruzi* infection increased with age and if they were Qom, but did not vary by gender (Table 2). The odds of infection in the total population significantly increased by 60% for each additional infected *T*. *infestans* collected in the domicile and by 40% with each additional infected co-inhabitant; increased with increasing household social vulnerability and decreased with increasing host availability index (Table 2).

**Table 2:** Generalized linear model (logit link function) of human seropositivity for *T*. *cruzi* infection for the total population (model 1) and children (model 2), Pampa del Indio, Argentina.

*Model 1*: *population model*				*Model 2*: *children*			
Variable	RI	POR (CI_95_)	P	Variable	RI	POR (CI_95_)	P
**Age (years)**	1	1.07 (1.06–1.08)	<0.001**	**Age (years)**	1	1.1 (1.04–1.3)	0.008*
**Infected vector abundance (1)**	1	1.6 (1.2–2.3)	0.003*	**Infected vector abundance (1)**	1	2.2 (1.001–4.8)	0.04*
**Social vulnerability (2)**	1	1.5 (1.2–1.8)	<0.001**	**Social vulnerability (2)**	0.9	1.3 (0.8–2.1)	0.2
**Interaction (1)** * **(2)**	1	0.7 (0.6–0.9)	0.01*	**Interaction (1)** * **(2)**	0.9	0.5 (0.2–0.98)	0.04*
**Host availability**	1	0.7 (0.6–0.9)	0.003*	**Host availability**	0.9	0.5 (0.2–0.9)	0.02*
**Number of seropositive co-inhabitants**	1	1.4 (1.3–1.6)	<0.001**	**Seropositive mother**	0.9		
**Ethnic group**	0.9			No		1	
Creole		1		Yes		3.8 (1.4–11)	0.009*
Qom		2.3 (1.1–4.6)	0.02*	**Gender**	0.7		
**Gender**	0.4			Male		1	
Male		1		Female		2 (0.96–4.1)	0.06~
Female		0.8 (0.6–1.1)	0.2	**Number of seropositive co-inhabitants**	0.6	1.3 (0.96–1.9)	0.08~
				**Recent insecticide spraying (2006–2008)**	0.5		
				No		1	
				Yes		0.5 (0.2–1.3)	0.2
				**Ethnic group**	0.3		
				Creole		1	
				Qom		1.4 (0.2–12.5)	0.7

** p < 0.001;

* 0.001 ≤ p ≤ 0.05;

^~^ 0.5 < p <1

POR: Prevalence odds ratio; RI: relative importance of the variable; CI_95_: 95% confidence interval

In the risk model for children, the positive effect of infected domestic vector abundance and the negative effect of host availability still held, but social vulnerability was no longer significant (Table 2). Also, the ethnic group did not have a significant effect on child infection, possibly because only one of the 70 seropositive children was creole. Including the mother’s seropositivity status (which was significantly and positively associated with child infection) led to a marginal effect of the number of seropositive co-inhabitants (Table 2). The recent partial insecticide spraying (over 2006–2008) did not have an effect on child infection status (Table 2); it rather had a protective effect if the abundance of infected vectors was removed from the model (POR = 0.4, CI_95_ = 0.1–0.9, p = 0.03). In both models, we observed a significant negative interaction between social vulnerability and infected vector abundance (Table 2). This indicates that the effect of infected vector abundance on *T*. *cruzi* human infection was lower in households with higher social vulnerability, suggesting people in households with high social vulnerability and low vector abundance have a similar infection risk as those in less vulnerable households but with higher vector abundance.

In both models, VIF < 2 indicated no multicollinearity issues. The infection risk model for the total population had AUC = 0.83, with a sensitivity of 83.6% and a specificity of 72.8% (S6 Fig). In a post-hoc classification performance analysis, the model predicted 19.3% of false positives and 4.7% of false negatives compared to the observed serosurveys’ results. However, the model fit the data poorly according to the Hosmer-Lemeshow test (p < 0.001), perhaps due to the lack of saturation of the model or a non-linear relationship between variables. By contrast, the risk model in children had a good fit to the data (Hosmer-Lemeshow test, p = 0.6); the AUC was 0.84 (S6 Fig), and it had higher sensitivity (87.5%) and lower specificity (68.4%) than the previous model. It predicted 28.8% of false positive cases but only 1.1% of false negatives compared to the observed serosurveys’ results. Moreover, the child infection model had a better classification performance (H-index: 0.423) than the total population infection model (H-index: 0.388), considering that misclassifying seropositive children as seronegative would be more costly (in terms of missing opportunities for case detection and treatment) than the misclassifying seronegative children.

**Fig 6:**
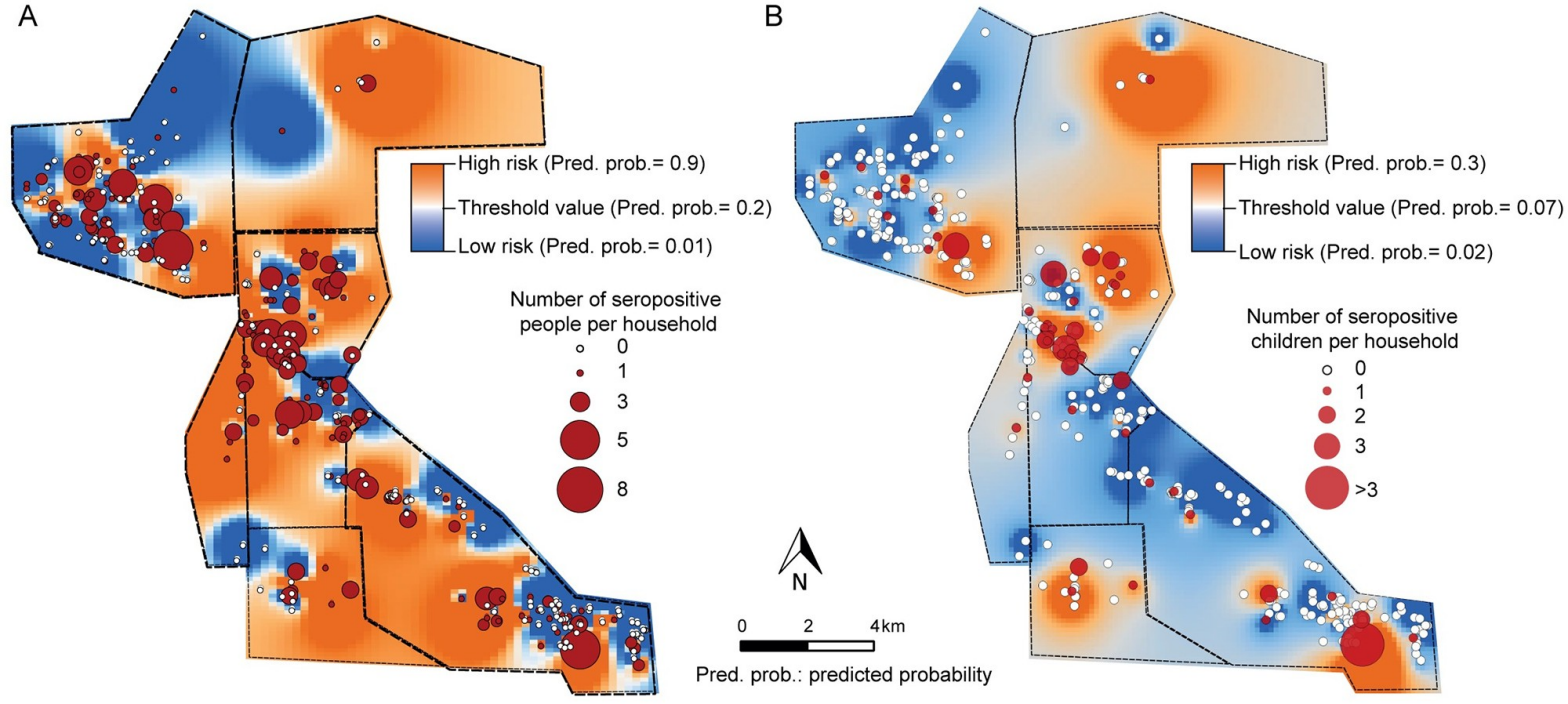
Risk maps of *T*. *cruzi* human infection for the total population (A) and children (B) in 2008, prior to a community-wide insecticide spraying campaign, Pampa del Indio, Argentina. The maps were created in QGIS 2.18.11. based on the data collected within the scope of this study (S4 Table).

The risk maps derived from the interpolation of the risk model documented the heterogeneous distribution of human infection and the occurrence of high-risk areas, which were more widespread when the total population was considered (Fig 6A) compared to the children risk map (Fig 6B). Although they failed to include a few households with seropositive children, most households with human cases were encompassed in these high-risk areas.

## Discussion

This study shows the multivariate relationship among the ecological, demographic and socio-economic factors affecting human *T*. *cruzi* infection in endemic rural communities with active vector-borne transmission. To the best of our knowledge, this may be the first study to comprehensively assess their combined effects and interactions on human *T*. *cruzi* infection and report significant effects of socio-economic position after adjusting for ecological factors. We also corroborated the effects of demographic variables (age, ethnic group, among others) and the relative abundance of infected *T*. *infestans* on human infection risk [16]. The odds of individual human infection significantly increased by 60% for each additional infected vector collected by unit of effort in the domicile, and by 40% with each infected co-inhabitant; increased with increasing household social vulnerability (a multidimensional index of poverty), and decreased with increasing domestic host availability (numbers of indoor-resting chickens or mammal hosts). A novel finding was the negative interaction between infected-vector abundance and social vulnerability, which indicates that members of more vulnerable households had an increased infection prevalence even at low infected-vector abundances. Household mobility was also a significant modifier of the association between vector exposure and human infection: local internal mobility reduced the effects of domiciliary infestation, possibly due to movers having less consistent exposures over time. Although transmission was clustered by household, the spatial analysis showed that transmission risk was heterogeneous within the study area, with hotspots of human and vector infection matching areas of higher social vulnerability.

Similar to other NTDs, the highly focal distribution of Chagas disease cases is determined by particular combinations of various ecological and social determinants [70,71]. The baseline (2008) human seroprevalence here estimated (29%) was considerably lower than that registered in adjacent rural communities (40%) of Pampa del Indio [16]; in other indigenous (59–71%) and creole (40–62%) communities living in more disadvantaged, isolated areas of Chaco province known as “The Impenetrable” [9,10,13], and in the Bolivian Chaco (40–80%) [29,72]. Our seroprevalence estimate was similar to other estimates (28–31%) registered elsewhere in the Argentine Chaco [8,11]. This heterogeneous distribution of human infection within Chaco province was evident in the 1980s when the intensity of vector-borne transmission was much higher [73], and remains evident almost three decades later. To date, the Gran Chaco eco-region remains as a hotspot for Chagas disease and other NTDs that disproportionally affect vulnerable rural communities and certain demographic groups [3,70].

The heterogeneous distribution of human infection was also captured through spatial analysis. Human cases, especially among children, were aggregated (Fig 5, S3 Fig) and matched the spatial aggregation of house infestation [41], vector infection and high social vulnerability (this paper). This pattern is consistent with vector-borne transmission mainly occurring at a household level [26,74], as human infection status was mostly explained by household-level factors. Household clustering of cases was both revealed when we included the household as a random variable, and when the number of infected co-inhabitants was included in the human risk model. In the child infection model, household clustering was closely linked to the mother’s seropositivity status (allowing for putative congenital transmission), and to being under similar vector exposures [27,74].

The strong and positive association between human infection and the abundance of *T*. *cruzi*-infected vectors indicated active vector-borne transmission prior to the community-wide insecticide spraying campaign we implemented. The occurrence of at least one seropositive child per household was significantly and positively associated with the abundance of infected *T*. *infestans* in the domicile, consistent with a more recent infection. Compared to the only previous study that quantified the effect of infected-vector domestic abundance on child infection risk, the magnitude we found was half of that estimated in rural communities of the dry Argentine Chaco two decades earlier (i.e., a two- versus four-fold increase per each additional infected vector) [26]. However, the partial insecticide spraying conducted within two years before the baseline vector survey, coupled with intense household mobility patterns [54] and housing conditions improving at a very slow pace [33], likely deflated the quantitative relationship between human and vector infection. Other studies reported a significant positive association between human infection and the presence of at least one infected domestic triatomine in rural communities [16,28,74], but not in a periurban community where child infection was instead associated with peridomestic infection in *T*. *infestans* [27]. In our study, most of the infestations occurred in the domicile where human-vector contact with *T*. *infestans* is most likely to occur, as the housing units only had a few peridomestic sites [41].

Host availability is an ecological variable (i.e., number and density of host-feeding sources) closely related to household size. Here, we used a summary index to represent domestic host availability because usually there is a positive and significant correlation between household size and the number of domestic dogs or cats and chicken indoors [e.g., 54]. The occurrence of at least one seropositive child was associated positively with the domestic host availability index, as children were more likely to occur in larger households; if other dimensions of risk are equal, there would be a greater chance of detecting at least one infected child in a larger household in which more members were screened. However, when the household number of infected (human) co-inhabitants was added to the individual risk model, the host availability index shifted from exerting positive effects to negative effects on human infection prevalence (weaker among children). Although there were no multicollinearity issues, understanding the effects of each type of host on the risk of human infection is hindered by the positive correlation between household size and the number of non-human domestic hosts, which pools key domestic reservoir hosts of *T*. *cruzi* (dogs and cats) with insusceptible hosts (chickens). Even though the host availability index exerted negative effects on human infection prevalence, this result needs to be interpreted with caution since household size or domestic host abundance sometimes, but not always, were positively associated with larger domestic vector abundance [19,75]. The species identity of non-human hosts is indeed a key factor: one study found that child infection prevalence increased with the household number of dogs but decreased with the number of chickens indoors [26], whereas in another study human infection prevalence increased only with 2 or more infected dogs per household [16]. Unfortunately, our study lacks information on the distribution of *T*. *cruzi* infection in dogs and cats.

The household social vulnerability index used as a surrogate of socio-economic status represents a multidimensional measure of poverty, which we found associated with the domestic abundance of infected *T*. *infestans* [54]. Herein, we also found significant and positive effects of social vulnerability on human infection prevalence. To this aim we elaborated a more comprehensive measure of socio-economic status since previous studies have focused on a few proxy variables: the goat-equivalent index [16], domicile’s characteristics (i.e., construction materials, size, cracks in walls, etc.) [26,29,30], and educational level [76]. The social vulnerability index summarized all of the above and also included household overcrowding, agricultural practices, at least one member with a stable source of income by employment, and the household number of welfare recipients [54]. The social vulnerability index also modified the effects of infected-vector abundance on human infection risk: the magnitude of this effect decreased as social vulnerability increased. This could be explained by several non-exclusive reasons: (i) the social vulnerability index in part may indicate a past, undetected vector infestation prior to the baseline survey in 2008 (i.e., past vector exposure); (ii) households with higher social vulnerability had greater local mobility [54], suggesting putative vector exposures elsewhere before the insecticide spraying campaign, and (iii) households with higher social vulnerability may allow more frequent human-vector contact given the smaller domicile and greater overcrowding. Social vulnerability may also affect household resilience to face house infestation and the consequences of vector exposure. The access of vulnerable households to health services (for diagnosis and treatment, for example) was lower; they were less likely to use domestic insecticides [54], possibly due to economic constraints, and depended on government-sponsored vector control actions.

Household-level variables combined with ethnic group and age determined variations in infection prevalence within the population. Ethnic group was a key demographic factor in stratifying infection risk; Qom people had a two-fold higher odds of being seropositive compared to creoles. Indigenous communities in the Argentine Chaco sometimes displayed higher infection rates than creole communities [9,13], but this pattern was not verified in rural communities in Pampa del Indio with a majority of creoles [16]. The effects of ethnic group on human infection, after adjusting for other social and ecological factors that co-vary with ethnicity, may be explained by differential mobility patterns between Qom and creole households [33,54]. The intense household mobility and the high rates of house turnover among the Qom most likely exerted negative impacts on *T*. *infestans* populations [41]. Movers were likely exposed to lower, more variable domestic infestations over time, and also had higher social vulnerability and a higher probability of occupying an infested house at baseline (preintervention) [54,77]. Complex vector exposure patterns created by human mobility were captured by the statistically significant interaction between domiciliary infestation and mobility in the child infection risk model. Although infection prevalence was not significantly different between movers that resided in infested *versus* non-infested houses at baseline, among non-movers infection peaked in children residing in infested houses, indicating prolonged vector exposure. The impact of human mobility was also evident when the aggregated spatial distribution of human cases at baseline (2008) was compared with the random pattern recorded in 2012–2015. This particular trait of the Qom population needs to be considered when designing vector control actions and disease screening strategies, given that vector indices alone would only detect a fraction of the *T*. *cruzi*-seropositive children with internal mobility.

Levy et al. (2007) [27] proposed a two-step screening strategy for *T*. *cruzi*-infected children based on an individual infection risk model adapted to periurban areas of Arequipa (Peru) with a history of recent vector-borne transmission. Their best model included age, *T*. *infestans* abundance in the domicile, and presence of at least one infected vector. They proposed first to screen children by their age and domestic vector abundance, followed by screening children living in neighboring houses up to the distance of spatial aggregation [27]. The model proposed in this study had a higher diagnostic ability (AUC = 0.84 vs 0.72) and high sensitivity, which would detect most of the infected children. Serological screening strategies that also include household socio-economic status and demographic characteristics, and if possible, the mother’s seropositivity status, are expected to perform better at the expense of added cost in obtaining such data. Such costs may be deflated by using this information for integrated interventions across NTDs.

One limitation of this study was the temporal lag between the baseline vector survey (2008) and the human serosurveys (2012–2015), which required to retrospectively assign the examined individuals to their 2008 residential location. Thus, missing data for human infection increased as a result of considerable human mobility, biasing the final infection model to non-mover individuals. Assignment errors were minimized by combining data from multiple sources and checking them with local primary healthcare agents; this led to the successful assignment of 80% of the population recorded in 2015. Human mobility patterns were only registered in 2012–2015; we assumed that this pattern was approximately constant over time (i.e., movers in 2012–2015 also behaved as movers in 2008) based on the observation that movers did it more than once over the study period [54]. Further considerations arise from the limitations of some of the tests used: the Hosmer-Lemeshow goodness-of fit test used to assess model fit has known limitations when applied to large sample sizes and cannot be considered alone to judge the validity of the model [78]. Thus, we also assessed its classification performance using the AUC and related metrics (i.e., sensitivity and specificity), and the H-index. While the risk model for the total population fitted the data poorly as opposed to the risk model for children, both models had a similar classification performance (AUC). The global spatial analyses are also limited by the number of neighbors at short distances: Despite we set the minimum distance to account for at least 3 neighbors on average, it may have not been sufficient to detect a spatial bivariate correlation between human and vector infection.

Lastly, the association between human infection and the explanatory variables could be affected by the temporality of events and rates of change over time, given that *T*. *cruzi* is a chronic infection acquired at an early age in endemic areas. However, this is not exactly so in resource-constrained rural areas such as in the Chaco, where transmission conditions changed little or not at all over extended periods in which no sustained control interventions were implemented, as recorded many times (e.g. [79]: the protective effects of interventions rapidly wanes and the system returns to its previous equilibrium), in which case the differences between groups would remain approximately invariant over relevant time periods. This is also why we focused on the model restricted to children <15 years of age (and compared the outcome with the general population model), as *T*. *cruzi* infection classically occurs at an early age in endemic areas (i.e., child infection is more closely associated to immediate pre-intervention conditions). The same rationale has been used to estimate the force of infection in humans (particularly children) and domestic dogs from endemic and periurban areas using cross-sectional data (e.g. [23,29,80,81]). The force of infection indicates the per unit time probability that a susceptible host becomes infected [82], and thus is a measure of incidence.

### Implications for disease control

The London Declaration Goals launched in 2012 proposed milestones to eliminate NTDs by 2020, including Chagas disease [83]. Access to diagnosis and treatment is one of the extant challenges for sustainable control of Chagas disease in endemic areas [50,83]. Chagas disease national estimates, resonated by WHO [1], may fail to approximate the true magnitude of the problem [83]. Integrating the ecological and social determinants of human infection with the spatial component allowed us to identify individuals with higher odds of being seropositive and households and geographic sectors with higher infection prevalence and presumably higher risk of infection. This information is key to design cost-effective serological screening strategies in resource-constrained areas for treatment of *T*. *cruzi*-infected people. Although our analysis took advantage of the detailed data collected during household surveys and census, the same approach could be done using national census data, though probably at the coarser spatial resolution that the data would allow.

Our infection risk model was also translated into an actionable tool (i.e., risk maps) that can point to higher-priority sections for targeted interventions oriented to suppress house (re)infestations, treat infected children, and thus reduce the burden of future disease. Given that Chagas disease is a chronic infection, in the context of effective vector surveillance and control but limited access to treatment, preintervention conditions can still guide the identification of high-risk households subject to an increased risk of reinfestation and transmission (e.g., [79,84,85]). Here the risk map would contribute to targeted surveillance and case detection, although human mobility can alter the extant patterns to a certain degree as shown in this study. This approach may also be useful for integrated control interventions across NTDs. Identifying the common eco-social determinants of other infectious diseases affecting the local population, such as geohelminthiases, TB and syphilis, can help improve the logistics and cost-effectiveness of interventions.

## Supporting information

S1 Text Checklist of STROBE recommendations for observational studies. (DOC)

S2 Text Performance of the two-tier ELISA serological tests and results for the total population residing in the study area between 2012 and 2015. (DOCX)

S1 Table Generalized linear mixed model of seropositivity for *T*. *cruzi* infection vs. demographic variables in 2008, clustered by household (logit link function). (DOCX)

S2 Table Generalized linear mixed model of seropositivity for *T*. *cruzi* infection vs. demographic and vector indices in 2008, clustered by household (logit link function) for the total population and for children. (DOCX)

S3 Table Generalized linear mixed model of child seropositivity for *T*. *cruzi* infection in 2008 clustered by household (logit link function), Pampa del Indio, Argentina. The prevalence odds ratio (POR) of the interaction terms indicate if the effect of infestation for non-movers increased (>1) or decreased (<1) in other mobility categories (out-migrants or return migrants).
(DOCX)

S4 Table Individual-level and household-level database including data on human infection with *Trypanosoma cruzi*, domiciliary infestation with *Triatoma infestans*, and environmental and socio-demographic variables in Area III, Pampa del Indio. (XLSX)

S1 Fig Flow diagram of the study design, vector control activities and the number of people tested in each phase of the serosurvey in 2012–2015 in Area III, Pampa del Indio. (TIF)

S2 Fig The 2012–2015 mid-period total population per 5-year age group and the population serologically tested for *T*. *cruzi* infection (number and percentage) in Pampa del Indio, Argentina. (TIF)

S3 Fig Predicted probabilities for the GLMM model of child seropositivity for *T*. *cruzi* infection regarding the interaction between domiciliary infestation and mobility patterns (non-movers vs. movers), Pampa del Indio, Argentina. (TIF)

S4 Fig Results from the global spatial point pattern analysis for the human and vector infection in 2008. (A) Univariate global analysis for the occurrence of at least one seropositive person in the household; (B) Variogram of the number of seropositive people per household; (C) Univariate global analysis for the occurrence of at least one seropositive children in the household; (D) Variogram of the number of seropositive children per household; (E) Univariate global analysis for the occurrence of at least one infected *T*. *infestans* considering all houses; (F) Univariate global analysis for the occurrence of at least one infected *T*. *infestans* considering only infested houses; (G) Bivariate global analysis for the correlation between children seropositivity and *T*. *infestans* infection; (H) Variogram for the spatial correlation between the abundance of infected *T*. *infestans* and the number of seropositive children per household. The lines with red dots indicate the observed values and the solid lines indicate the confidence ‘envelopes’. For the qualitative-mark global analysis we present the L(r) estimated by the Ripley weighted K-function, in which r represents the distance in meters. For the quantitative-mark global analysis we present the rho(r) (mark variogram), which indicates if neighboring households present similar mark values (a lower rho value means more similar values) evaluated at each distance r.
(TIF)

S5 Fig Variograms from the global spatial point pattern analysis for socio-demographic variables and human or vector infection: The social vulnerability index vs. the number of seropositive people per household (A), vs. the number of seropositive children (B), and vs. the abundance of infected *T*. *infestans* (C); and the host availability index vs. the number of seropositive people per household (E), vs. the number of seropositive children (F), and vs. the abundance of infected *T*. *infestans* (G). The lines with red dots indicate the observed values and the solid lines indicate the confidence ‘envelopes’.
(TIF)

S6 Fig Receiver operating characteristic curves (ROC) of the *T*. *cruzi* infection risk models for the total population (A) and children (B), with their respective the optimum threshold value for classification (i.e. maximizes sensitivity and specificity) and 95% confidence intervals. (TIF)
