# Supplementary material for: Human Trypanosoma cruzi infection is driven by eco-social interactions in rural communities of the Argentine Chaco
Source: PLoS Negl Trop Dis. 2019 Dec 16;13(12):e0007430. doi: 10.1371/journal.pntd.0007430 (PMC6936860; doi:10.1371/journal.pntd.0007430)
Supplement: S1 Text — (DOC) [file pntd.0007430.s001.doc]

STROBE Statement—Checklist of items that should be included in reports of ***cross-sectional studies***

The sections and excerpts of the manuscript where the items can be found are in italic.

|  | Item No | Recommendation |
| --- | --- | --- |
| **Title and abstract** | 1 | (*a*) Indicate the study’s design with a commonly used term in the title or the abstract  *In abstract: The seroprevalence of T. cruzi among 1,929 local residents examined in a cross-sectional study.* |
|  |  | (*b*) Provide in the abstract an informative and balanced summary of what was done and what was found  *In abstract: Using generalized linear mixed models, the risk of human infection increased by 60% with each additional infected triatomine and by 40% with each seropositive household co-inhabitant; increased significantly with increasing household social vulnerability (a multidimensional index of poverty), and decreased with increasing host availability in sleeping quarters. A significant negative interaction between household social vulnerability and the relative abundance of infected T. infestans indicated that vulnerable household residents were exposed to a higher risk of infection even at low infected-vector abundances. Household mobility within the study area reduced the effects of domiciliary vector abundance, possibly due to less consistent exposures. Nonetheless, the seroprevalence rates of movers and non-movers were not significantly different. Human infection was clustered by household and at a larger spatial scale, with hotspots of human and vector infection matching areas of higher social vulnerability*. |
| Introduction | | |
| Background/rationale | 2 | Explain the scientific background and rationale for the investigation being reported  *Introduction, paragraph 1: The transmission of zoonotic and vector-borne diseases is an “inherently ecological process” involving intraspecific and interspecific interactions between vector, pathogen and host populations. As shown by Ross-Macdonald style mathematical models, the transmission of vector-borne pathogens is determined by the density and survival of insect vectors, vector-host contact rate, host susceptibility, vector and host infectivity, and duration of infection. When human populations are implicated, pathogen transmission dynamics also involve socio-economic, cultural, political, psychological and ethical factors, which pertain to the human dimension of disease.*  *Introduction, paragraph 3: In the Gran Chaco, human infection with T. cruzi usually occurs within sleeping quarters before reaching 15 years of age and is transmitted by domiciliary Triatoma infestans. Most studies of human infection with T. cruzi in endemic areas have focused on the seroprevalence distribution among demographic subgroups and/or on the effects of vector presence, abundance and T. cruzi infection status. Although the association between human T. cruzi infection and selected socio-demographic factors has been investigated, these studies either did not address the combined effects of ecological and social variables due to limited data availability, or only considered a few socio-demographic variables.* |
| Objectives | 3 | State specific objectives, including any prespecified hypotheses  *Introduction, paragraph 5: As part of a broader research and control program on the transmission of T. cruzi in rural communities of the Argentine Chaco, this study investigated the effects of household-level vector indices and demographic factors on human T. cruzi infection at individual, household and community levels. We also considered the household socio-economic status and the interaction between social and ecological variables using two indices of household social vulnerability as a measure of socio-economic inequalities and of host availability in the domicile. Both indices were positively associated with the abundance of infected domiciliary vectors. Herein, we hypothesize that the interaction between ecological and social factors would enhance the risk of T. cruzi transmission to humans, which cannot solely be explained by variation in vector indices.* |
| Methods | | |
| Study design | 4 | Present key elements of study design early in the paper  *See first section: “study design” (Methods, paragraphs 3 and 4)* |
| Setting | 5 | Describe the setting, locations, and relevant dates, including periods of recruitment, exposure, follow-up, and data collection  *For location see section “study area” (Methods, paragraphs 1 and 2)*  *For relevant dates:*  *Methods, paragraph 1: This area was subjected to a vector control and Chagas disease research program initiated in 2008 with a follow-up period of 7 years as of 2015.*  *In October 2008, a baseline vector survey found that a third of the inhabited houses were infested with T. infestans, mainly within human sleeping quarters, and virtually all (93.4%) houses were sprayed with insecticides immediately after.*  *Methods, paragraph 3: This study integrated vector and epidemiological information collected during the 2008-2015 period.*  *Methods, paragraph 4: Four years after community-wide insecticide spraying and with a low risk of vector-borne transmission (<1% of infested houses), we conducted a series of serosurveys aimed at total population coverage (older than 9 months old) during the 2012-2015 period.* |
| Participants | 6 | (*a*) Give the eligibility criteria, and the sources and methods of selection of participants  *Methods, paragraph 4: Using a participatory approach, we had meetings with the local population to discuss project tasks and coordinate activities with local healthcare agents and hospital personnel. During 2012-2013, people were recruited passively through the radio, schools and word of mouth; venipuncture was done at schools and at the primary health posts of each large community to draw a blood sample of 3-7 ml. In April 2015, recruitment was done actively and blood draws were conducted coupled to the vector survey. The protocol is described in detail elsewhere.* |
| Variables | 7 | Clearly define all outcomes, exposures, predictors, potential confounders, and effect modifiers. Give diagnostic criteria, if applicable  *Diagnostic criteria (Methods, paragraph 5): Serum samples were tested using two different ELISA tests based on conventional (Chagatest, Wiener) and recombinant antigens (ELISA Rec V3.0, Wiener) as described elsewhere. Discordant results between both ELISAs were resolved via an indirect immunofluorescence antibody test conducted at the reference center for Chagas disease serodiagnosis (Instituto Nacional de Parasitología Dr M. Fatala Chaben). A person was considered seropositive (i.e., infected) if at least two tests were reactive.*  *For outcome, exposures, predictors and potential confounders see section “Infection risk model” (Methods, paragraphs 12 and 13)* |
| Data sources/ measurement | 8* | For each variable of interest, give sources of data and details of methods of assessment (measurement). Describe comparability of assessment methods if there is more than one group  *See sections “serodiagnosis of human infection” (Methods, paragraph 6), “vector-related indices” (Methods, paragraph 7) and “epidemiological data pre-processing” (Methods, paragraph 8 and 9).* |
| Bias | 9 | Describe any efforts to address potential sources of bias  *Methods, paragraph 10: For each variable we checked whether the missing values were missing completely at random by building a dummy variable (missing and non-missing values) and analyzing the significance of Spearman’s correlation coefficient with any another independent variable in the data set. Most of the variables with missing values (collected through household surveys) were missing completely at random, except for educational level and overcrowding in 2008, in which the missing data corresponded to households that had moved or out-migrated by 2012 (the year when these data were collected). Missing data for human infection per household were also biased towards adult males (who refused to participate more often than other demographic groups), and movers, as we were not able to assign their residential location in 2008 to all of them. We excluded from the analysis 197 people tested who resided elsewhere but were visiting the study area at the time of the serosurvey.*  *Discussion, paragraph 9: Missing data for human infection increased as a result of considerable human mobility. Assignment errors were minimized by combining data from multiple sources and checking with local primary healthcare agents, which led to a successful assignment of 80% of the population recorded by 2015. Human mobility patterns were only registered in 2012-2015; we assumed that this pattern was constant over time (i.e., movers in 2012-2015 also behaved as movers in 2008) based on the observation that movers did it more than once.* |
| Study size | 10 | Explain how the study size was arrived at  *Methods, paragraph 2: The population in the area monotonically increased during the 2008-2015 period, from 2,392 people and 407 inhabited houses in 2008 to 2,548 people and 587 houses in 2015.*  *Methods, paragraph 4: For the serosurveys in 2012-2015, we aimed at total population coverage (older than 9 months old).* |
| Quantitative variables | 11 | Explain how quantitative variables were handled in the analyses. If applicable, describe which groupings were chosen and why  *See section “epidemiological data pre-processing” (Methods, paragraph 8 and 9).* |
| Statistical methods | 12 | (*a*) Describe all statistical methods, including those used to control for confounding  *Methods, paragraph 11: For all proportions, 95% confidence intervals (CI_95_) were estimated using the Agresti and Coull method if sample sizes were greater than 50, and the Wilson method for smaller sample sizes. We used χ^2^ tests for bivariate analysis of categorical variables; generalized linear models (GLM) with logit link function when the outcome of interest had a binary distribution, and a negative binomial GLM (link function: log) when analyzing count data, such as the number of seropositive people. In the case of binary response variables, the relative risk was expressed as Odds Ratios (OR), and in the case of count data as incidence rate ratios (IRR). Negative binomial regression was preferred to Poisson regression given the overdispersed distributions. Mixed-effects models (GLMM) were also considered when individual data was analyzed to account for possible household-related random effects.*  *Spatial analysis (Methods, paragraph 14): Global point pattern analysis (univariate and bivariate) were performed for human and vector infection using the weighted K-function implemented in Programita. Random labeling was used to test the null hypothesis of random occurrence of events among the fixed spatial distribution of all households. We used quantitative and qualitative labels for each household as previously described. Monte Carlo simulations (n = 999) were performed and the 95% ‘confidence envelope’ was calculated with the 2.5% upper and lower simulations. Additionally, local spatial analysis on the abundance of (infected) vectors were performed using the G* statistic implemented in PPA.* |
|  |  | (*b*) Describe any methods used to examine subgroups and interactions  *Methods, paragraph 12: The infection risk model was estimated for the entire population and for children separately. We compared a GLMM model (logit link function) considering the household as a random variable and a GLM model in both cases.*  *Methods, paragraph 13: We used an information theoretic approach and Akaike’s information criterion (AIC) to identify the best-fitting models given the data collected, and a multimodel inference approach to account for model selection uncertainty using the MuMin R-package. This approach was used to identify the ecological and social factors associated with house infestation, as described elsewhere. Odds Ratios (ORs) and their 95% confidence intervals were calculated from model-averaged coefficients.*  *Multicollinearity was assessed by the Variance Inflation Factor (VIF), and model fitting for the logistic regression was assessed by the Hosmer-Lemeshow goodness of fit test and the Receiver Operator Curve (ROC), including the area under the ROC (AUC)* |
|  |  | (*c*) Explain how missing data were addressed  *Methods, paragraph 13: Multimodel inference do not allow for missing data, therefore individuals with missing data were not considered in the models.* |
|  |  | (*d*) If applicable, describe analytical methods taking account of sampling strategy  *Methods, paragraph 11: GLMM models were chosen because of individuals were exposed to the same household-level effects.* |
|  |  | (*e*) Describe any sensitivity analyses  *Methods, paragraph 13: The relative importance (RI) of each variable is defined as the sum of Akaike weights in each model in which the variable is present; RI takes values from 0 to 1. To assess sensitivity and specificity of the models, we employed an optimal threshold value that minimized the sum of error frequencies. This value was obtained by finding the maximum sum of sensitivity and specificity for all threshold values t (sens(t)+spec(t)) using the pROC R-package.* |
| Results | | |
| Participants | 13* | (a) Report numbers of individuals at each stage of study—eg numbers potentially eligible, examined for eligibility, confirmed eligible, included in the study, completing follow-up, and analysed  *Results, paragraph 1: During 2012 and 2015, we tested 1,929 people that resided in the study area at the time of the serosurveys. Passive recruitment during 2012-2013 at local schools and primary health posts led to a coverage of 47.8% of the local population, which increased to 77% during 2015, when active recruitment of participants was coupled with vector surveys (S1 Fig). Diagnosis coverage was highest in school children (80.9%) and adult women (76.8%) in general (Fig 1).* |
|  |  | (b) Give reasons for non-participation at each stage  *S1 Figure: Local residents refused to participate, moved out of the area or we were not able to draw sufficient blood for the serosurvey.* |
|  |  | (c) Consider use of a flow diagram  *S1 Figure* |
| Descriptive data | 14* | (a) Give characteristics of study participants (eg demographic, clinical, social) and information on exposures and potential confounders  *Results, paragraph 3: We were able to assign the local residency status as of 2008 (i.e., their house identification code in 2008) to 82.6% of the people tested. The overall seroprevalence estimated for 2008 was 29% (CI_95_ = 26.7-31.4, n = 1373). It increased significantly with age from 5.7% in children younger than 5 y.o. to 25% in teenagers (14 to 19 y.o.), then jumped to 50% in young adults (20 to 29 y.o.), and remained around 60% in older adults (Fig 2). Although females had a lower overall seroprevalence rate than males (26.5 vs 31.6%; χ^2^ test, df =1, p = 0.04), this difference was more evident in adults (Fig 2) and was not significant after adjusting for age (S1 Table). The seroprevalence for Qom people almost doubled that observed for creoles (29.7 vs 18.7%; χ^2^ test, df = 1, p = 0.02) (S1 Table).* |
|  |  | (b) Indicate number of participants with missing data for each variable of interest  *This information can be derived from S1 Table.* |
| Outcome data | 15* | Report numbers of outcome events or summary measures  *Results, paragraph 2: The two-tiered ELISA serological testing showed almost perfect agreement between tests (kappa index = 0.9, p < 0.001). Seropositive results for T. cruzi infection were observed in 25.3% of the samples, whereas 5.5% that were discordant later tested negative at the reference laboratory. Only 1.5% (CI_95_ = 4*10^-4^ - 3.0%) of children born after the community-wide insecticide spray in 2008 were seropositive. All four T. cruzi-seropositive children born post-intervention had T. cruzi-seropositive mothers and no vectors were collected in their domiciles during the surveillance phase. For the same age group (children <6 y.o.), the risk of infection was higher if they had been born before the community-wide spraying (logistic regression, OR_2008_ = 6.2, CI_95_ = 1.2-31.8, p = 0.01), after adjusting for age.* |
| Main results | 16 | (*a*) Give unadjusted estimates and, if applicable, confounder-adjusted estimates and their precision (eg, 95% confidence interval). Make clear which confounders were adjusted for and why they were included  *See Table 1 and Table 2* |
|  |  | (*b*) Report category boundaries when continuous variables were categorized  *Not applicable* |
|  |  | (*c*) If relevant, consider translating estimates of relative risk into absolute risk for a meaningful time period  *Not applicable* |
| Other analyses | 17 | Report other analyses done—eg analyses of subgroups and interactions, and sensitivity analyses  *Results, paragraph 15: In both models, VIF<2 indicated no multicollinearity issues. The infection risk model for the total population had AUC = 0.83, with a sensitivity of 83% and a specificity of 72% (Fig 7). In a post-hoc classification, the model predicted 19.3% of false positives and 4.7% of false negatives. The model fit the data poorly according to the Hosmer-Lemeshow test (p < 0.001), perhaps due to the lack of saturation of the model, or non-linear relationship between variables, or limits to the goodness-of-fit test due to a large number of cases. By contrast, the risk model in children had a good fit to the data (Hosmer-Lemeshow test, p = 0.6); the AUC was 0.84 (Fig 7), and it had higher sensitivity (87%) but lower specificity (68%) compared to the previous model. It predicted 28.8% of false positive cases but only 1.1% of false negatives.* |
| Discussion | | |
| Key results | 18 | Summarise key results with reference to study objectives  *Discussion, paragraph 1* |
| Limitations | 19 | Discuss limitations of the study, taking into account sources of potential bias or imprecision. Discuss both direction and magnitude of any potential bias  *Discussion, paragraph 9* |
| Interpretation | 20 | Give a cautious overall interpretation of results considering objectives, limitations, multiplicity of analyses, results from similar studies, and other relevant evidence  *Discussion, paragraphs 3, 4 and 5.* |
| Generalisability | 21 | Discuss the generalisability (external validity) of the study results  *Discussion, paragraphs, 2, 7, 10.* |
| Other information | | |
| Funding | 22 | Give the source of funding and the role of the funders for the present study and, if applicable, for the original study on which the present article is based  *See funding section.* |

*Give information separately for exposed and unexposed groups.

**Note:** An Explanation and Elaboration article discusses each checklist item and gives methodological background and published examples of transparent reporting. The STROBE checklist is best used in conjunction with this article (freely available on the Web sites of PLoS Medicine at http://www.plosmedicine.org/, Annals of Internal Medicine at http://www.annals.org/, and Epidemiology at http://www.epidem.com/). Information on the STROBE Initiative is available at www.strobe-statement.org.
