# Supplementary material for: Human Trypanosoma cruzi infection is driven by eco-social interactions in rural communities of the Argentine Chaco
Source: PLoS Negl Trop Dis. 2019 Dec 16;13(12):e0007430. doi: 10.1371/journal.pntd.0007430 (PMC6936860; doi:10.1371/journal.pntd.0007430)
Supplement: S2 Text — (DOCX) [file pntd.0007430.s002.docx]

**S2 Text.** *Performance of the two-tier ELISA serological tests and results for the total population residing in the study area between 2012 and 2015.*

The two-tiered ELISA serological testing showed almost perfect agreement between tests (kappa index = 0.9, p < 0.001). Seropositive results for *T. cruzi* infection were observed in 25.3% of the samples, whereas 5.5% that were discordant later tested negative at the reference laboratory. Only 1.5% (CI_95_ = 4*10^-4^ - 3.0%) of children born after the community-wide insecticide spray in 2008 were seropositive. All four *T. cruzi*-seropositive children born post-intervention had *T. cruzi*-seropositive mothers and no vectors were collected in their domiciles during the surveillance phase. For the same age group (children <6 y.o.), the risk of infection was higher if they had been born before the community-wide spraying (logistic regression, POR_2008_ = 6.2, CI_95_ = 1.2-31.8, p = 0.01), after adjusting for age. Only 1.4% of the people tested had been exposed to *T. infestans* due to transient domiciliary reinfestations during the surveillance period, but all 38 also lived in infested houses prior to the 2008 insecticide campaign.
