# Supplementary material for: Human Trypanosoma cruzi infection is driven by eco-social interactions in rural communities of the Argentine Chaco
Source: PLoS Negl Trop Dis. 2019 Dec 16;13(12):e0007430. doi: 10.1371/journal.pntd.0007430 (PMC6936860; doi:10.1371/journal.pntd.0007430)
Supplement: S1 Table — (DOCX) [file pntd.0007430.s003.docx]

**S1 Table.** Generalized linear mixed model of seropositivity for *T. cruzi* infection vs. demographic variables in 2008, clustered by household (logit link function).

| **Variables** | **Seroprevalence (CI_95_)** | **N** | **OR (CI_95_)** | **P** |
| --- | --- | --- | --- | --- |
| **Age group** |  |  |  |  |
| Younger than 5 y.o. | 5.7 (3.7-8.8) | 332 | 1 |  |
| 5 to 9 | 11.2 (8.0-15.5) | 285 | 2.8 (1.3-6.1) | 0.01* |
| 10 to 14 | 15.0 (10.9-20.3) | 226 | 4.9 (2.2-10.6) | <0.001** |
| 15 to 19 | 25.0 (18.9-32.2) | 164 | 6.8 (3.0-15.7) | <0.001** |
| 20 to 24 | 46.3 (38.1-54.8) | 136 | 39.7 (17.3-91.11) | <0.001** |
| 25 to 29 | 50.4 (41.3-59.6) | 113 | 32.6 (13.9-76.5) | <0.001** |
| 30 to 34 | 57.0 (46.3-67.1) | 86 | 67.8 (26.4-173.9) | <0.001** |
| 35 to 39 | 54.8 (42.3-66.8) | 62 | 81.1 (29.3-224.8) | <0.001** |
| 40 to 44 | 60.9 (50.5-70.3) | 92 | 116.7 (45.6-298.7) | <0.001** |
| 45 to 49 | 65.5 (51.9-76.9) | 55 | 134.5 (46.0-393.5) | <0.001** |
| 50 to 54 | 55.6 (39.1-70.9) | 36 | 68.8 (21.0-226.1) | <0.001** |
| 55 and older | 58.1 (46.6-68.8) | 74 | 121.1 (44.0-332.9) | <0.001** |
| **Gender** |  |  |  |  |
| Male | 31.6 (28.2-35.2) | 668 | 1 |  |
| Female | 26.5 (23.4-29.9) | 706 | 0.8 (0.5-1.1) | 0.1 |
| **Ethnic group** |  |  |  |  |
| Creole | 18.7 (11.9-28.0) | 91 | 1 |  |
| Qom | 29.7 (27.2-32.2) | 1283 | 3.7 (1.5-9.1) | <0.01* |
| *** p<0.001; * 0.001≤p≤0.05; ~ 0.5<p<1* | | | | |
