# Supplementary material for: Human Trypanosoma cruzi infection is driven by eco-social interactions in rural communities of the Argentine Chaco
Source: PLoS Negl Trop Dis. 2019 Dec 16;13(12):e0007430. doi: 10.1371/journal.pntd.0007430 (PMC6936860; doi:10.1371/journal.pntd.0007430)
Supplement: S2 Table — (DOCX) [file pntd.0007430.s004.docx]

**S2 Table.** Generalized linear mixed model of seropositivity for *T. cruzi* infection vs. demographic and vector indices in 2008, clustered by household (logit link function) for the total population and for children.

|  |  | **Baseline vector survey** | | **Reported past exposure** | |
| --- | --- | --- | --- | --- | --- |
| **Model** | **Variables** | **OR (CI95)** | **P** | **OR (CI95)** | **P** |
| General population | **House infestation** |  |  |  |  |
|  | No | 1 |  | 1 |  |
|  | Yes | 2.3 (1.4-3.9) | 0.001** | 1.5 (1-2.2) | 0.06~ |
|  | **Age** | 1.09 (1.07-1.1) | <0.001** | 1.09 (1.07-1.1) | <0.001** |
|  | **Gender** |  |  |  |  |
|  | Male | 1 |  | 1 |  |
|  | Female | 0.8 (0.6-1.1) | 0.2 | 0.8 (0.6-1.1) | 0.2 |
|  | **Ethnic group** |  |  |  |  |
|  | Creole | 1 |  | 1 |  |
|  | Qom | 4.4 (1.8-11) | 0.001** | 4.3 (1.7-11) | 0.002** |
|  | **AIC (n)** | 1262 (1360) | | 1180 (1249) | |
| Children (<15 y.o.) | **House infestation** |  |  |  |  |
|  | No | 1 |  | 1 |  |
|  | Yes | 3.7 (1.3-10) | 0.01* | 0.9 (0.4-2.4) | 0.9 |
|  | **Age** | 1.16 (1.05-1.3) | 0.003* | 1.15 (1.03-1.3) | 0.01* |
|  | **Gender** |  |  |  |  |
|  | Male | 1 |  | 1 |  |
|  | Female | 1.5 (0.7-3.2) | 0.3 | 1.6 (0.7-3.5) | 0.3 |
|  | **Ethnic group** |  |  |  |  |
|  | Creole | 1 |  | 1 |  |
|  | Qom | 5.5 (0.3-112) | 0.3 | 5.5 (0.3-93) | 0.2 |
|  | **Seropositive mother** |  |  |  |  |
|  | No | 1 |  | 1 |  |
|  | Yes | 7.9 (2.5-25) | <0.001** | 9.7 (2.9-33) | <0.001** |
|  | **AIC (n)** | 289 (564) | | 276 (521) | |
| *** p<0.001; * 0.001≤p≤0.05; ~ 0.5<p<1* | | | | | |
