# Supplementary material for: Human Trypanosoma cruzi infection is driven by eco-social interactions in rural communities of the Argentine Chaco
Source: PLoS Negl Trop Dis. 2019 Dec 16;13(12):e0007430. doi: 10.1371/journal.pntd.0007430 (PMC6936860; doi:10.1371/journal.pntd.0007430)
Supplement: S3 Table — The prevalence odds ratio (POR) of the interaction terms indicate if the effect of infestation for non-movers increased (>1) or decreased (<1) in other mobility categories (out-migrants or return migrants). (DOCX) [file pntd.0007430.s005.docx]

**S3 Table.** Generalized linear mixed model of child seropositivity for *T. cruzi* infection in 2008 clustered by household (logit link function), Pampa del Indio, Argentina. The prevalence odds ratio (POR) of the interaction terms indicate if the effect of infestation for non-movers increased (>1) or decreased (<1) in other mobility categories (out-migrants or return migrants)*.*

| **Variables** | **POR (CI_95_)** | **P** |
| --- | --- | --- |
| **Infestation (non-movers)** |  |  |
| No | 1 |  |
| Yes | 6.1 (1.9-19.0) | 0.002* |
| **Age** | 1.14 (1.03-1.3) | 0.01* |
| **Gender** |  |  |
| Male | 1 |  |
| Female | 1.5 (0.7-3.2) | 0.3 |
| **Ethnic group** |  |  |
| Creole | 1 |  |
| Qom | 7.1 (0.4-139.0) | 0.2 |
| **Mother infection status** |  |  |
| No | 1 |  |
| Yes | 8.0 (2.5-25.0) | <0.001** |
| **Mobility (2012-2015)** |  |  |
| Non-movers (non-infested houses) | 1 |  |
| Movers (non-infested houses) | 1.8 (0.4-8.5) | 0.5 |
| Out-migrants (non-infested houses) | 2.2 (0.2-20.0) | 0.5 |
| Return migrants (non-infested houses) | 0 | 1 |
| **Interactions§** |  |  |
| Movers*infested houses | 0.10 (0.01-0.99) | 0.05* |
| Out-migrants*infested houses | 0 | 1 |
| Return migrants*infested houses | 0 | 1 |
| § The reference category is non-movers living in infested houses | | |
| *POR: Prevalence odds ratio; CI_95_: 95% confidence interval* | | |
| *** p < 0.001; * 0.001 ≤ p≤ 0.05; ~ 0.5 < p < 1* | | |
