# Supplementary figures and images for: Human Trypanosoma cruzi infection is driven by eco-social interactions in rural communities of the Argentine Chaco
Source: PLoS Negl Trop Dis. 2019 Dec 16;13(12):e0007430. doi: 10.1371/journal.pntd.0007430 (PMC6936860; doi:10.1371/journal.pntd.0007430)

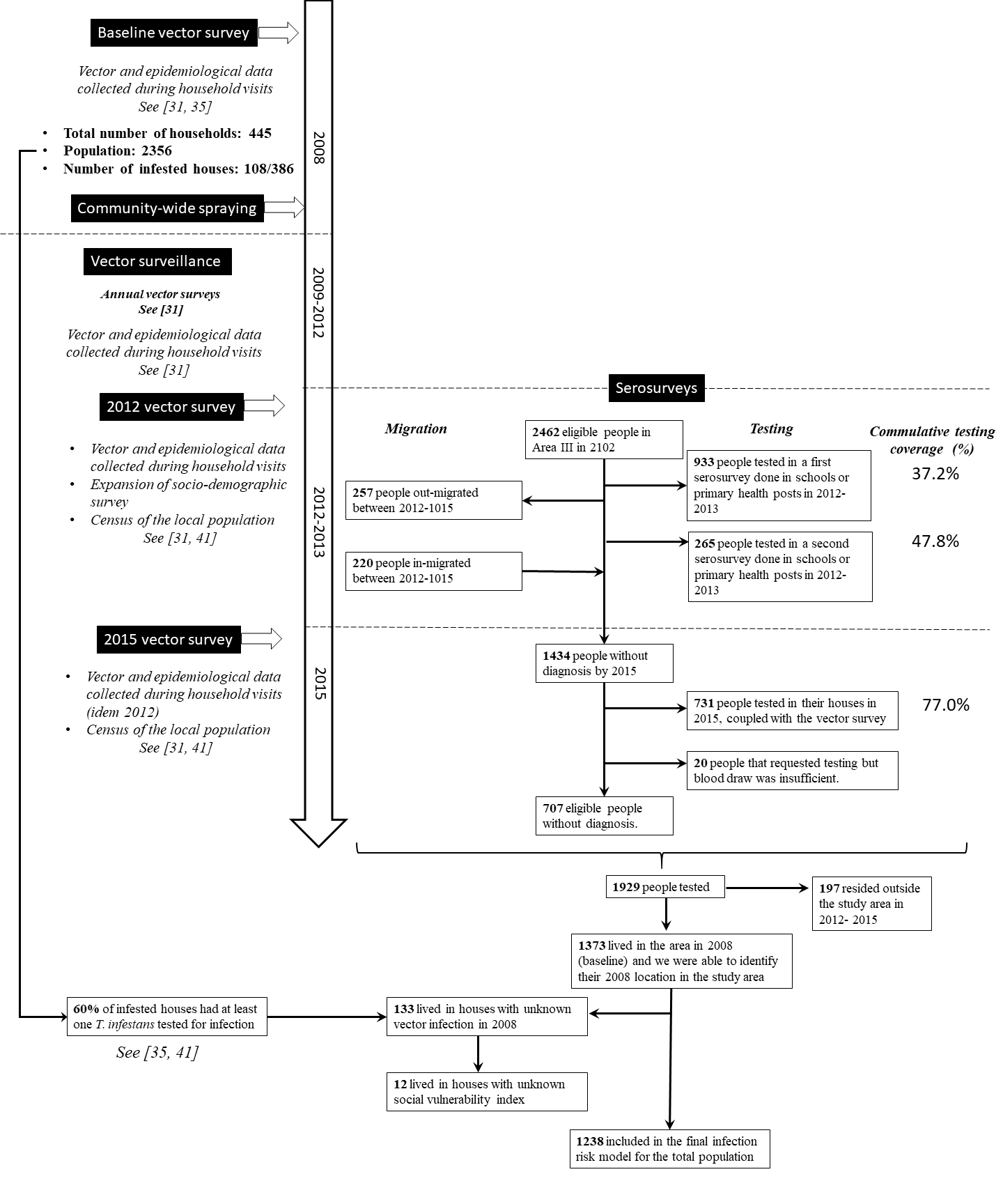

Supplement: S1 Fig — (TIF) [file pntd.0007430.s007.tif]

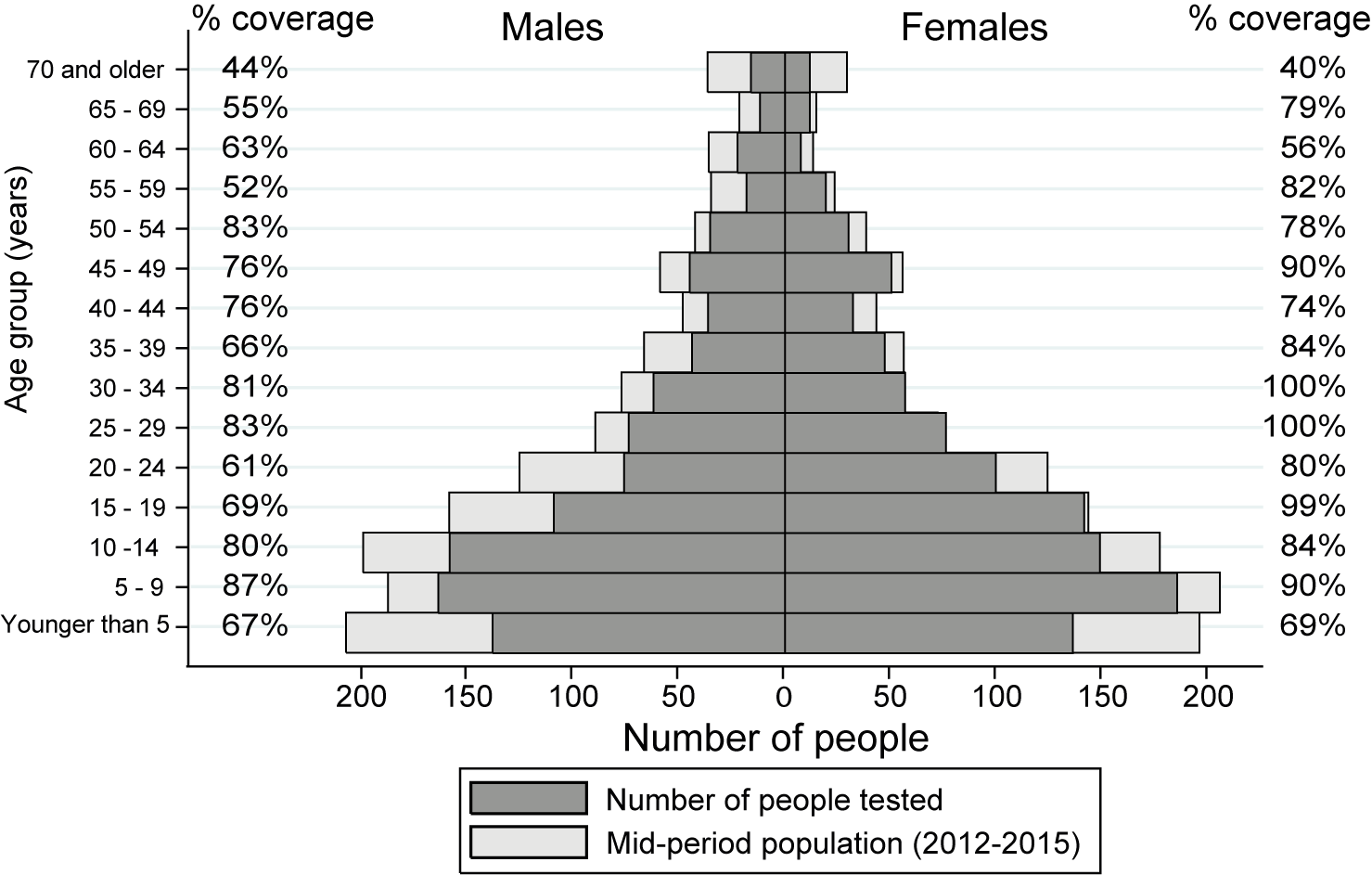

Supplement: S2 Fig — (TIF) [file pntd.0007430.s008.tif]

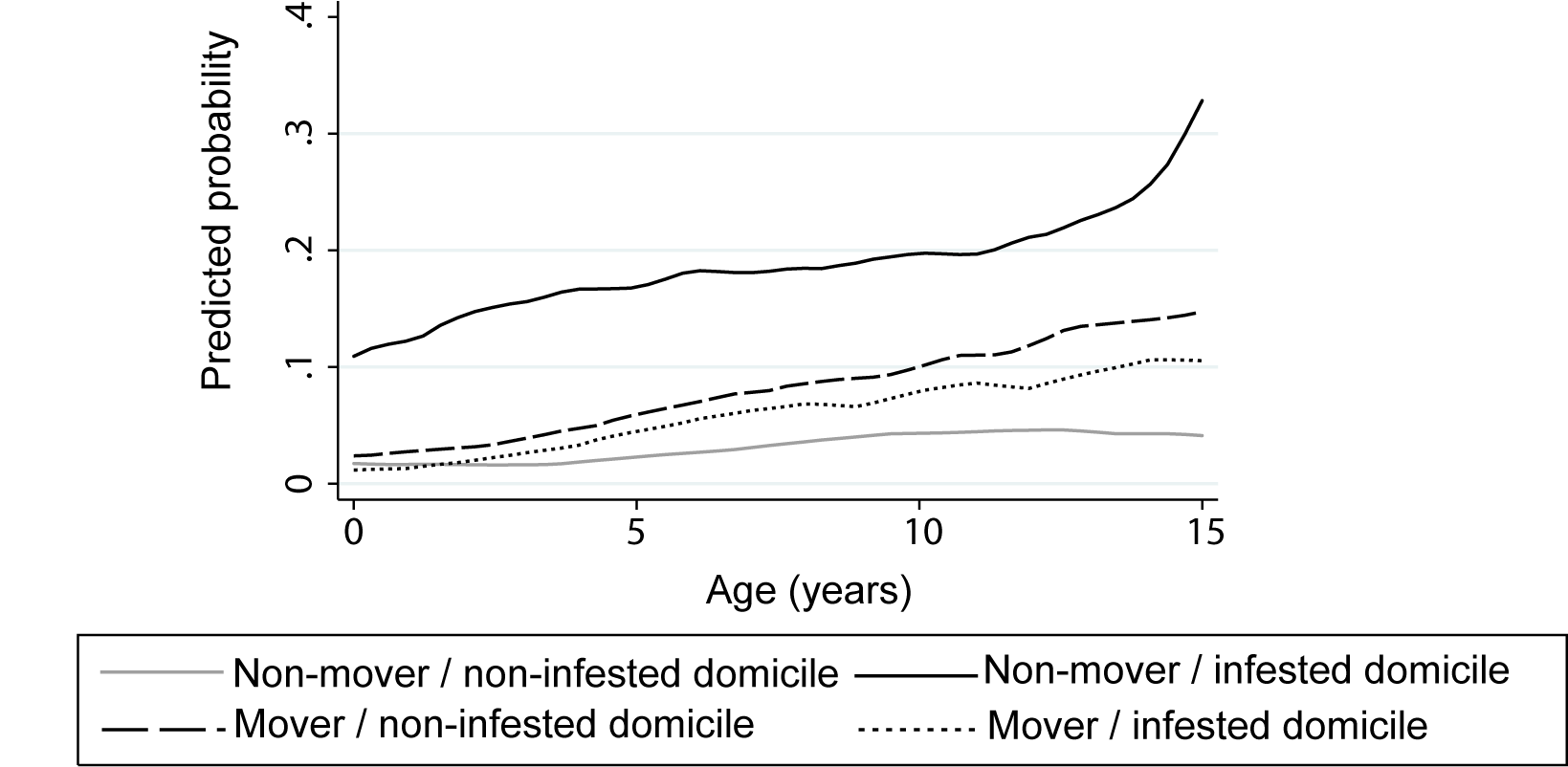

Supplement: S3 Fig — (TIF) [file pntd.0007430.s009.tif]

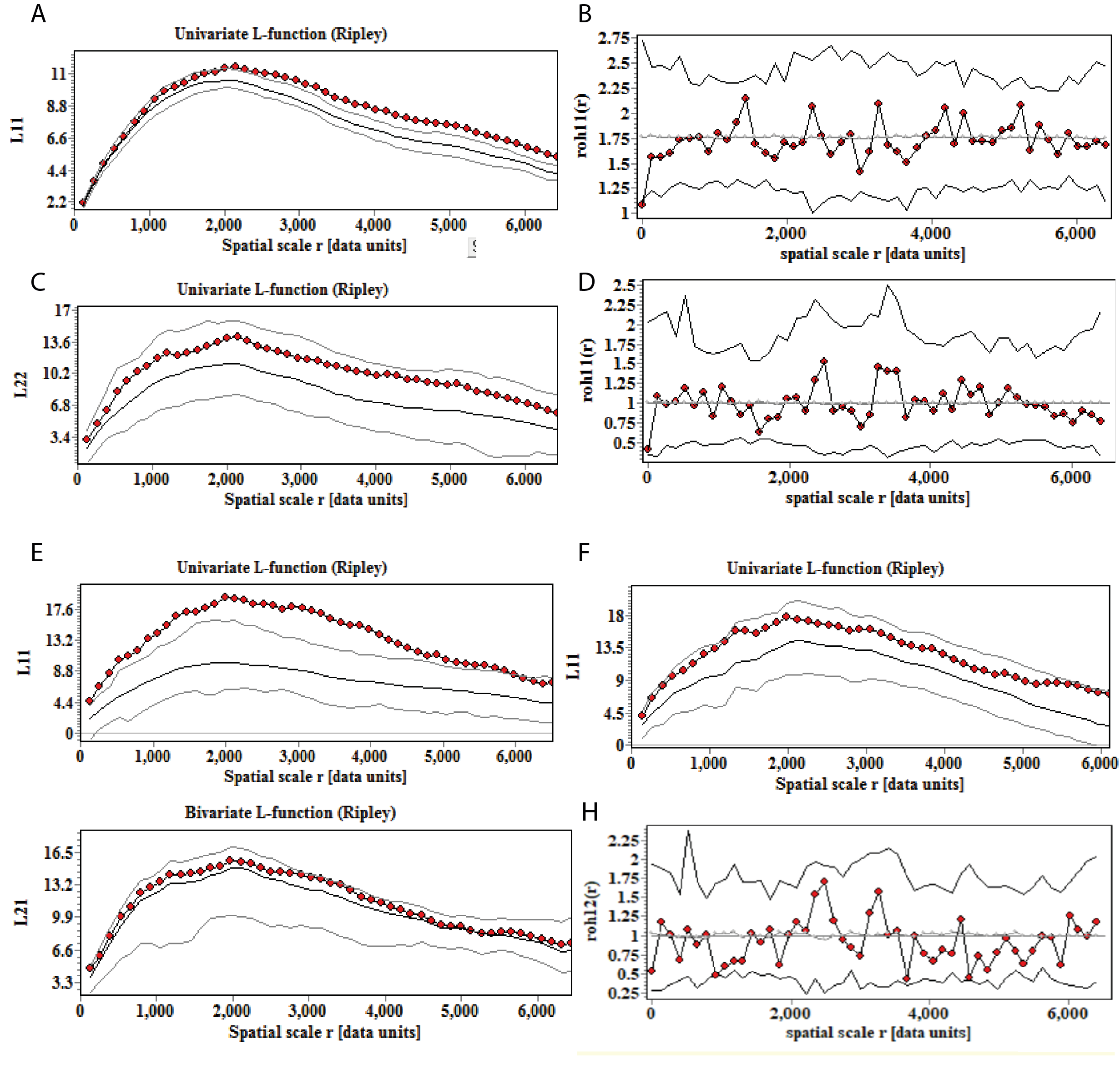

Supplement: S4 Fig — (A) Univariate global analysis for the occurrence of at least one seropositive person in the household; (B) Variogram of the number of seropositive people per household; (C) Univariate global analysis for the occurrence of at least one seropositive children in the household; (D) Variogram of the number of seropositive children per household; (E) Univariate global analysis for the occurrence of at least one infected T. infestans considering all houses; (F) Univariate global analysis for the occurrence of at least one infected T. infestans considering only infested houses; (G) Bivariate global analysis for the correlation between children seropositivity and T. infestans infection; (H) Variogram for the spatial correlation between the abundance of infected T. infestans and the number of seropositive children per household. The lines with red dots indicate the observed values and the solid lines indicate the confidence ‘envelopes’. For the qualitative-mark global analysis we present the L(r) estimated by the Ripley weighted K-function, in which r represents the distance in meters. For the quantitative-mark global analysis we present the rho(r) (mark variogram), which indicates if neighboring households present similar mark values (a lower rho value means more similar values) evaluated at each distance r. (TIF) [file pntd.0007430.s010.tif]

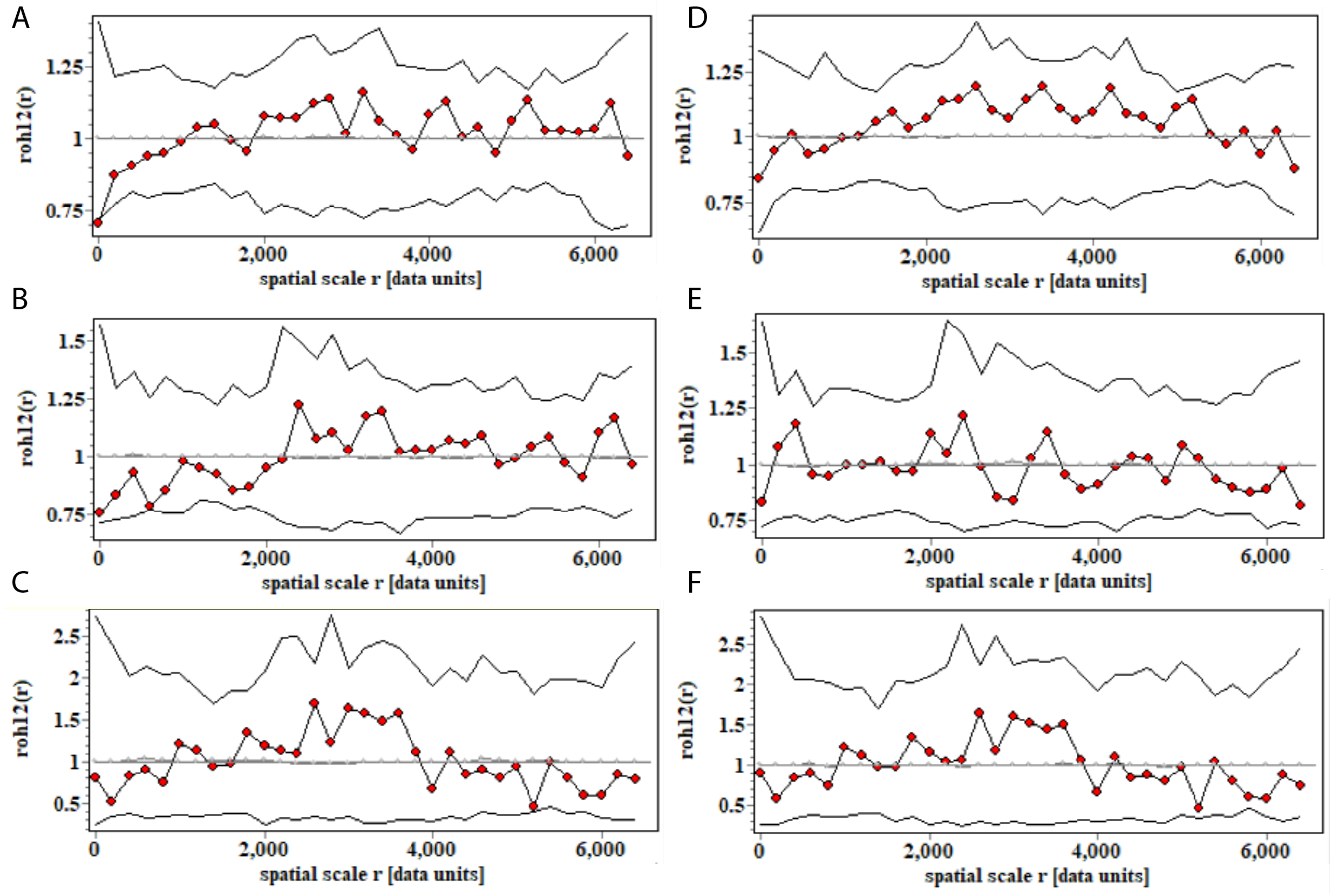

Supplement: S5 Fig — The lines with red dots indicate the observed values and the solid lines indicate the confidence ‘envelopes’. (TIF) [file pntd.0007430.s011.tif]

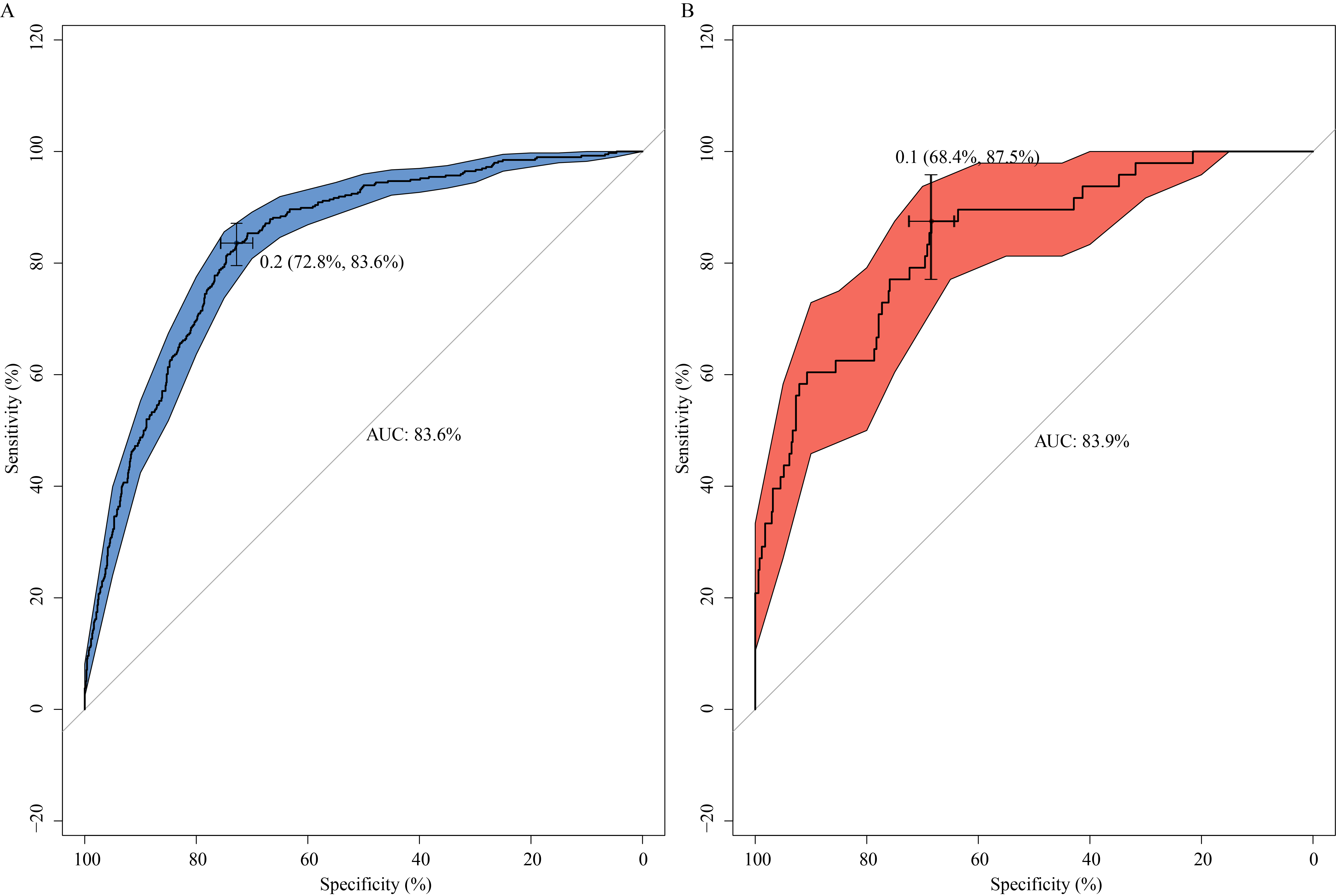

Supplement: S6 Fig — (TIF) [file pntd.0007430.s012.tif]
